# Magnetic nanoparticles enhance the cellular immune response of dendritic cell tumor vaccines by realizing the cytoplasmic delivery of tumor antigens

**DOI:** 10.1002/btm2.10400

**Published:** 2022-09-09

**Authors:** Linghong Huang, Zonghua Liu, Chongjie Wu, Jiansheng Lin, Ning Liu

**Affiliations:** ^1^ Department of Biomedical Engineering Jinan University Guangzhou China; ^2^ Department of Bone and Joint Surgery The First Affiliated Hospital of Jinan University, Jinan University Guangzhou China; ^3^ Department of Anatomy Hunan University of Chinese Medicine Changsha China

**Keywords:** antigen delivery, DC vaccines, magnetic nanoparticles

## Abstract

Dendritic cells (DCs)‐based tumor vaccines have the advantages of high safety and rapid activation of T cells, and have been approved for clinical tumor treatment. However, the conventional DC vaccines have some severe problems, such as poor activation of DCs in vitro, low level of antigen presentation, reduced cell viability, and difficulty in targeting lymph nodes in vivo, resulting in poor clinical therapeutic effects. In this research, magnetic nanoparticles Fe_3_O_4_@Ca/MnCO_3_ were prepared and used to actively and efficiently deliver antigens to the cytoplasm of DCs, promote antigen cross‐presentation and DC activation, and finally enhance the cellular immune response of DC vaccines. The results show that the magnetic nanoparticles can actively and quickly deliver antigens to the cytoplasm of DCs by regulating the magnetic field, and achieve cross‐presentation of antigens. At the same time, the nanoparticles degradation product Mn^2+^ enhanced immune stimulation through the interferon gene stimulating protein (STING) pathway, and another degradation product Ca^2+^ ultimately promoted cellular immune response by increasing autophagy. The DC vaccine constructed with the magnetic nanoparticles can more effectively migrate to the lymph nodes, promote the proliferation of CD8^+^ T cells, prolong the time of immune memory, and produce higher antibody levels. Compared with traditional DC vaccines, cytoplasmic antigen delivery with the magnetic nanoparticles provides a new idea for the construction of novel DC vaccines.

## 1 INTRODUCTION

With the huge therapeutic potential, cancer immunotherapy is expected to become the mainstream of cancer treatment. Tumor vaccines, as an important immunotherapy method, have attracted wide attention due to their strong specificity, low side effects, generation of immune memory to long‐term supervise the tumor metastasis, and recurrence.^1^, ^2^, ^3^ Like traditional vaccines, ordinary tumor vaccines containing tumor antigens/adjuvants can be directly immunized to hosts.^4^ However, it has been found that conventional tumor vaccines cannot be effectively taken up by antigen presenting cells (APCs) in the body, cannot effectively activate APCs, and fail to produce effective cellular immunity, resulting in poor therapeutic effects.^4^, ^5^, ^6^ To solve this problem, researchers isolate the patient's own dendritic cells (DCs) out of the body and treated them directly in vitro with tumor antigen/adjuvant complex to promote the uptake of the tumor antigen by the DCs. The DCs sensitized in this way are called DC vaccines. Compared with ordinary tumor vaccines, these DC vaccines have more advantages. First, vaccination with the DC vaccines that are sensitized in vitro will greatly save the time for DCs to take up antigen and mature in the body, and activate T cells more quickly and effectively. Second, it is easier for the DC vaccines to activate CD8^+^ T cells.^7^ Third, the safety of DC vaccines is higher.^8^ At present, four DC tumor vaccines have been approved for marketing in the world. Among them, the DC vaccine (Provenge) was first approved by the Food and Drug Adminisrtation (FDA) for the treatment of prostate cancer in 2010.^9^

Although many DC vaccines have entered clinical trials, the clinical response effectiveness of patients is still low. For example, the objective clinical response rate of the DC vaccines for prostate cancer and renal cell carcinoma is only 7.7%–12.7%.^10^, ^11^ The main reasons for the inefficiency of DC vaccines include: poor activation of DCs in vitro, reduced activity and migration ability of DCs due to long‐term in vitro culture. As a result, the DC vaccines loaded with antigens usually stay at the injection site for a long time and are eliminated within 48 h.^12^, ^13^ These problems hinder the clinical application of DC vaccines. In order to improve the efficacy of DC vaccines, researchers have made various attempts to improve the clinical treatment effect of DC vaccine. For example, Toll‐like receptor (TLR) agonists (lipopolysaccharide, polyI:C, Resiquimod, etc.) or inflammatory cytokines (tumor necrosis factor‐α [TNF‐α], IL‐1β, IL‐6, etc.) are used to activate DCs in vitro.^14^ Fusion of DCs and tumor cells into DC–tumor hybrid cells can produce stronger cellular immunity, compared with the mixture of DCs and tumor cells.^15^ Downregulation of the expression of cytokine signal transduction inhibitor 1 in DCs using siRNA can enhance the immunotherapy effect of DC vaccines.^16^ Using the CD40 ligand expressed by T or B cells to bind to CD40 on the surface of DCs to increase expression of their costimulatory molecules can increase the efficacy of the DC vaccines.^17^ Using physical techniques such as electroporation can promote the transfection of mRNA to DCs and enhance their antigen presentation.^18^ Although these methods increase the anti‐tumor immune response of DC vaccines to a certain extent, there are still problems to be solved, such as complicated preparation technology, low preparation success rate, high cost, and low response rate. Therefore, there is an urgent need to develop easily prepared, low‐cost, efficient, and safe DC vaccines.

In recent years, magnetic nanoparticles have been widely used in tumor diagnosis, treatment, and imaging because of their good biocompatibility, stable performance, easy surface modification, and magnetic controlled drug delivery and release.^19^, ^20^, ^21^, ^22^ For example, Chiang et al.^22^ used magnetic nanoparticles to load checkpoint inhibitor and T‐cell activators, which could target to tumors by applying an external magnetic field to achieve in situ expansion of tumor‐infiltrating T cells and repair the immunosuppressive tumor microenvironment. Inspired by the directional movement of magnetic nanoparticles, we propose to use magnetic nanoparticles to deliver tumor antigens to the DC cytoplasm under magnetic field, promote antigen cross‐presentation, activate the DCs at the same time, and thereby enhance the cellular immune response. In addition, as reported, Mn^2+^ can activate cyclic guanosine monophosphate adenosine monophosphate synthase‐interferon gene stimulating protein (cGAS‐STING) cascade reaction to induce antigen cross‐presentation of DCs,^23^, ^24^ and Ca^2+^ can regulate autophagy, which will help increase antigen cross‐presentation.^25^, ^26^, ^27^ This stimulated research's interest in Mn/Ca based bioactive materials and adjuvants. For example, calcium phosphate,^28^, ^29^, ^30^ calcium carbonate,^27^, ^31^, ^32^ nano manganese (Mn^2+^‐cyclic guanosine monophosphate‐adenosine monophosphate synthase (cGAMP) coordinated nano vaccine,^33^ Mn^2+^‐cyclic dinucleotide (CDN) particles,^34^ and MnFe_2_O_4_ nanoparticles^35^) were used to enhance the immune response of antigens.

In this work, we prepared magnetic nanoparticles Fe_3_O_4_@Ca/MnCO_3_ with Fe_3_O_4_ nanoparticles as the core and Ca/MnCO_3_ as the shell, and used the nanoparticles to construct a new type of DC vaccines with multiple advantages. First, magnetic field can wirelessly control the magnetic nanoparticles to actively and quickly reach the cytoplasm of DCs. Second, the surface modification of Fe_3_O_4_ nanoparticles with degradable Ca/MnCO_3_ coating can alleviate the aggregation problem of Fe_3_O_4_ nanoparticles and meanwhile enhance their loading capacity. Third, Ca/MnCO_3_ coating and Fe_3_O_4_ particles have the merits of simple preparation, low cost, and good bio‐safety, and can be used for safe inoculation.^36^ Fourth, the Ca/MnCO_3_ coating can enhance immune stimulation effect, because its degradation product Mn^2+^ has vaccine adjuvant effect,^23^, ^24^ and its degradation product Ca^2+^ can regulate autophagy^25^, ^26^ and also promote antigen cross‐presentation.^27^ The magnetic nanoparticles‐based antigen delivery system provides a new strategy for the development of novel DC vaccines.

## 2 METHODS

### 2.1 Materials

Calcium chloride (CaCl_2_), sodium carbonate (Na_2_CO_3_), manganese chloride monohydrate (MnCl_2_·H_2_O), and Fe_3_O_4_ nanoparticles were bought from Macklin (Shanghai, China). Cell culture medium 1640 RPMI, fetal bovine serum and penicillin–streptomycin were obtained from Gibco (CA, USA). Lysotracker Green DND‐26 was purchased from ThermoFisher (Waltham, USA). Fluo‐4 AM kit, Bicinchoninic acid (BCA) protein detection kit, 2‐(4‐amidinophenyl)‐6‐indolecarbamidine dihydrochloride (DAPI) staining solution and red blood cell lysis buffer were obtained from Beyotime (Shanghai, China). The cell counting kit‐8 (CCK‐8) was obtained from Dojindo (Kyushu, Japan). Monodansylcadaverine (MDC) was obtained from Solarbio (Beijing, China). Fluorescein isothiocyanate (FITC)‐Tunnel cell apoptosis detection kit and CD8‐GB13429 were obtained from Servicebio (Wuhan, China). LumiKine Xpress mIFN‐β 2.0 enzyme linked immunosorbent assay (ELISA) kit was purchased from InvivoGen (CA, USA). Murine granulocyte‐macrophage colony‐stimulating factor (GM‐CSF) and murine Interleukin (IL) 4 were purchased from PeproTech (NJ, USA). All flow cytometry antibody dyes and cytokine detection ELISA kits were purchased from BioLegend (CA, USA). All female C57BL/6 mice (4–6 weeks) used in the research were purchased from Beijing HFK Laboratory Animal Technology Co. (Beijing, China). In addition, all animal experiments were approved by the Institute of Laboratory Animal Science of Jinan University and complied with animal ethics and guidelines of Jinan University.

### 2.2 Fabrication and characterization of Fe_3_O_4_ @Ca/MnCO_3_ nanoparticles

First, CaCl_2_ and MnCl_2_ mixed solutions (0.016 M, the molar ratio of Ca^2+^ and Mn^2+^ = 1:1) were prepared with glycerol/water solution (1/1, v/v). Then, Fe_3_O_4_ nanoparticles (5 mg) were added to CaCl_2_ and MnCl_2_ mixed solution (10 ml) and stirred for 30 min. NH_4_HCO_3_ (10 ml, 0.16 M) in glycerol/water solution (1/1, v/v) was then added to the mixture and stirred for 1 h at 50°C. Then, the precipitate was centrifuged and washed with water three times. Finally, the formed nanoparticles were subject to characterization with scanning electron microscope (SEM, Zeiss, Germany), transmission electron microscope (TEM, JEM‐2010HR, Japan), Fourier infrared spectrometer (FT‐IR, VERTEX70, Germany), laser nanoparticle sizer (Malvern, Britain), X‐ray powder diffractometer (XRD, Miniflex 600, Rigaku, Japan), vibrating sample magnetometer (LakeShore, USA), and specific surface area and porosity analyzer (ASAP 2460, Micromeritics, USA).

### 2.3 Adsorption and release of OVA by Fe_3_O_4_ @Ca/MnCO_3_ nanoparticles

Firstly, 1 ml of OVA solution (300 μg/ml) was prepared with physiological saline. Then, the Fe_3_O_4_ (5 mg) and Fe_3_O_4_@Ca/MnCO_3_ nanoparticles (5 mg) were added into the ovalbumin (OVA) solution (1 ml). The resulting suspensions were stirred at room temperature, and the free OVA in the solution was detected at different time points with the BCA kit. Then, Fe_3_O_4_@Ca/MnCO_3_/OVA were placed in acid phosphate buffered saline (PBS) buffer solution (pH 5.6), and BCA kits were used to detect the release behavior of OVA in vitro acidic environment.

### 2.4 Isolation and stimulation of bone marrow‐derived dendritic cells

Bone marrow‐derived dendritic cells (BMDCs) were obtained from the healthy female C57BL/6 mice. Briefly, the bone marrow cells were obtained from tibias and femurs, and red blood cells in it were lysed with red blood cell lysate. These bone marrow cells were cultured with RPMI1640 complete medium (containing 20 ng/ml GM‐CSF and 10 ng/ml IL‐4) and seeded into 6‐well plates. The medium was replaced in every 2 days. On the 6th day, the immature BMDCs were seeded into 24‐well low attachment surface plates (1 × 10^5^ cells/well) and treated for 24 h with OVA, Fe_3_O_4_@Ca/MnCO_3_ + OVA (mixture of soluble OVA and blank nanoparticles), Fe_3_O_4_@Ca/MnCO_3_/OVA (Fe_3_O_4_@Ca/MnCO_3_ loaded with OVA, without magnetic field), Fe_3_O_4_@Ca/MnCO_3_(M) (Fe_3_O_4_@Ca/MnCO_3_ loaded with OVA, with magnetic field created by neodymium iron boron permanent magnetic disks) or Alum/OVA formulations (5 μg OVA/well). Then, the fluorescent dye‐labeled antibodies solutions (anti‐CD11c‐APC, anti‐H_2_K_b_/SIINFEKL [MHC I]‐PE, anti‐MHC II‐PE, anti‐CD80‐FITC, anti‐CD40‐PerCP‐Cy5.5, and anti‐CD86‐PerCP‐Cy5.5) were used to detect the expression of OVA‐specific class I major histocompatibility complex (MHC I), MHC II, CD80, CD86, and CD40 molecules on CD11c^+^ DCs. The cells were detected by a flow cytometer (Beckman Coulter, USA). Meanwhile, the secretion levels of interferon (IFN)‐β in the supernatants were detected with mIFN‐β 2.0 ELISA kits as detailed in the manufacturer's instructions.

In addition, the levels of BMDC maturation and antigen presentation induced by Fe_3_O_4_/OVA and Fe_3_O_4_@Ca/MnCO_3_/OVA group were compared. Firstly, in order to ensure consistent OVA loading amount on Fe_3_O_4_ and Fe_3_O_4_@Ca/MnCO_3_ nanoparticles, 120 μg OVA was added into 5 mg of Fe_3_O_4_ or Fe_3_O_4_@Ca/MnCO_3_ suspension to obtain Fe_3_O_4_/OVA and Fe_3_O_4_@Ca/MnCO_3_/OVA formulation. Then, the immature BMDCs were seeded into 24‐well low attachment surface plates (1 × 10^5^ cells/well) and treated for 24 h with OVA, Fe_3_O_4_/OVA, Fe_3_O_4_@Ca/MnCO_3_/OVA, or Alum/OVA formulations (5 μg OVA/well). Subsequently, the cells were stained with the above fluorescent dye‐labeled antibodies solutions and detected by the flow cytometer.

### 2.5 Cell uptake and subcellular co‐localization of magnetic nanoparticles

DC2.4 and BMDCs were seeded in glass‐bottom cell culture dishes (35 mm in diameter) (about 5 × 10^4^ cells/dish), and incubated for 24 h. Then, the cells were incubated at 37°C for 6 h with the OVA molecule labeled with Cy5.5 (Cy5.5‐OVA), Fe_3_O_4_@Ca/MnCO_3_ + Cy5.5‐OVA, Fe_3_O_4_@Ca/MnCO_3_/Cy5.5‐OVA, or Fe_3_O_4_@Ca/MnCO_3_/Cy5.5‐OVA(M) formulations. Subsequently, the cells were incubated for 2 h with the Lyso‐Green fluorescent dye solution, fixed for 20 min with the 4% paraformaldehyde solution, and then stained for 10 min with DAPI dye solution (300 μl). Finally, the cells were observed with a confocal laser scanning microscope (CLSM, LSM 880, Zeiss, Germany).

The DC2.4 cells were seeded in 24‐well plates (5 × 10^4^ cells/well), and incubated for 24 h at 37°C. The cells were incubated for 6 h at 37°C with PBS, Cy5.5‐OVA, Fe_3_O_4_@Ca/MnCO_3_ + Cy5.5‐OVA, Fe_3_O_4_@Ca/MnCO_3_/Cy5.5‐OVA, or Fe_3_O_4_@Ca/MnCO_3_/Cy5.5‐OVA(M) formulations. Finally, the cells were washed twice to remove the free particles, and detected by the flow cytometer.

### 2.6 Observation of intracellular Fe_3_O_4_ @Ca/MnCO_3_ nanoparticles in DC2.4

The DC2.4 cells were seeded in 10 cm petri dishes (5 × 10^5^ cells/dish) and incubated for 24 h. Subsequently, the cells were incubated for 24 h with Fe_3_O_4_@Ca/MnCO_3_ nanoparticles. Then, the cells were fixed for 15 min with 1 ml of glutaraldehyde solution. Next, the cells were gently collected and re‐fixed for 2 h with glutaraldehyde solution. Finally, the cells were embedded in resin, sliced, and observed by the TEM.

### 2.7 Detection of lysosome integrity

The DC2.4 cells were seeded in 24‐well plates (5 × 10^4^ cells/well) and incubated for 24 h. Next, the cells were stained for 1 h with 5 μg/ml acridine orange dye solution. After removing the dye, the cells were cultured for 24 h with PBS, OVA, Fe_3_O_4_@Ca/MnCO_3_ + OVA, Fe_3_O_4_@Ca/MnCO_3_/OVA, Fe_3_O_4_@Ca/MnCO_3_/OVA(M), or Alum/OVA formulations. The distribution of acridine orange in the cells was observed with a fluorescence microscope (DMRA2, Leica, Germany).

The DC2.4 cells were seeded in 96‐well black plates (1 × 10^4^ cells/well) and incubated for 24 h at 37°C. The treatment of acridine orange staining was as same as the above steps. Then, the red (620 nm) and green (485 nm) fluorescence intensities of the cells were detected with a multifunctional microplate reader (Cytation5, Biotek, USA). Lysosome integrity of the treated cells (%) = (red/green fluorescence intensity of the treated cells)/(red/green fluorescence intensity of the control cells) × 100%.

### 2.8 Detection of intracellular Mn^2+^ level and Ca^2+^

The DC2.4 cells were seeded in 6‐well plates (1 × 10^5^ cells/well) and incubated for 24 h at 37°C. Then, the cells were incubated at 37°C for 24 h with OVA, Fe_3_O_4_@Ca/MnCO_3_ + OVA, Fe_3_O_4_@Ca/MnCO_3_/OVA, or Fe_3_O_4_@Ca/MnCO_3_/OVA(M) formulations. Subsequently, the cells were lysed with 1 ml cell lysis buffer, then the supernatant was collected and diluted to 5 ml. Mn content in each sample was determined using inductively coupled plasma‐atomic emission spectrometry (iCAP 7000, Thermo, USA). For detection of intracellular Ca^2+^ level, the above cells were stained for 30 min with Flo‐4 AM staining solution (200 μl, 5 μmol/L) and detected with the flow cytometer.

### 2.9 Detection of autophagosome

The DC2.4 cells were seeded in 24‐well plates (5 × 10^4^ cells/well) and incubated for 24 h at 37°C. Then, the cells were incubated at 37°C for 24 h with OVA, Fe_3_O_4_@Ca/MnCO_3_ + OVA, Fe_3_O_4_@Ca/MnCO_3_/OVA, Fe_3_O_4_@Ca/MnCO_3_/OVA(M), or Alum/OVA formulations. Subsequently, the cells were stained for 1 h with MDC staining and observed with the fluorescence microscope.

### 2.10 Detection of IFN‐β

The BMDCs were seeded in 24‐well plates (5 × 10^4^ cells/well), and incubated at 37°C for 24 h with OVA, Fe_3_O_4_@Ca/MnCO_3_ + OVA, Fe_3_O_4_@Ca/MnCO_3_/OVA, Fe_3_O_4_@Ca/MnCO_3_/OVA(M), or Alum/OVA formulations. Subsequently, their supernatants were collected to detect the level of IFN‐β.

### 2.11 Coculture assays

The above‐activated BMDCs were cocultured with naive splenocytes in 24‐well plates at a splenocytes/BMDCs ratio of 1,000,000/100,000. Their supernatants were collected after 48 h and evaluated with ELISA for IFN‐γ, TNF‐α, IL‐4, IL‐6, and IL‐10. Meanwhile, the cells were dyed with the fluorescent dye‐labeled antibodies solutions (anti‐CD3‐APC and anti‐CD8‐PerCP‐Cy5.5) to detect the ratio of CD8^+^ T cells.

### 2.12 Detection of antigen migration to lymph nodes

First, the above activated BMDCs were collected and suspended in PBS at a final concentration of 1 × 10^7^ cells/ml. The C57BL/6 female mice (4–6 weeks old) were randomly divided into four groups (*n* = 4), and immunized with 100 μl of activated DCs. On the 2nd day after immunization, the lymph nodes of the mice were collected and observed with a small animal bioluminescence imaging system (IVIS Lumina III, PerkinElmer, USA). Subsequently, the lymphocytes were obtained and counted with the flow cytometer.

### 2.13 Immunohistochemical analysis

The female C57BL/6 mice (4–6 weeks old) were randomly divided into five groups (*n* = 5). Subsequently, the mice were subcutaneously immunized with 100 μl of the OVA‐activated DCs (1 × 10^6^ cells/mouse). On the 2nd and 7th day, the spleens and lymph nodes of the mice were collected, and fixed with 4% paraformaldehyde solution for further immunohistochemical analysis. The distributions of antigen protein OVA in spleens and lymph nodes were observed by an optical microscope (Leica DMI6000, Germany).

### 2.14 Immunization evaluations of the DC vaccines in vivo

The C57BL/6 female mice (4–6 weeks old) were randomly divided into five groups (*n* = 5) and subcutaneously immunized with 100 μl of the OVA, Fe_3_O_4_@Ca/MnCO_3_ + OVA, Fe_3_O_4_@Ca/MnCO_3_/OVA, Fe_3_O_4_@Ca/MnCO_3_/OVA(M), or Alum/OVA ‐treated DCs (1 × 10^6^ cells/mouse). The mice were vaccinated twice with interval of 7 days. On the 7th day after the 2nd vaccination, the sera and splenocytes were separated from the mice.

In addition, the immunological evaluation of Fe_3_O_4_/OVA or Fe_3_O_4_@Ca/MnCO_3_/OVA nanoparticles activated DCs was carried out in vivo. The C57BL/6 female mice (4–6 weeks old) were randomly divided into five groups (*n* = 5) and subcutaneously immunized with 100 μl of the OVA, Fe_3_O_4_/OVA, Fe_3_O_4_@Ca/MnCO_3_/OVA, or Alum/OVA‐treated DCs (1 × 10^6^ cells/mouse). The mice were vaccinated twice with interval of 7 days. On the 7th day after the 2nd vaccination, the sera, splenocytes, and lymphocytes were separated from the mice.

### 2.15 Determination of OVA‐specific antibodies in sera

The OVA‐specific antibodies in the sera were detected with ELISA. First, the antigen OVA solution (10 μg/ml) was prepared with carbonate buffer (0.1 M, pH = 9.6) and added to 96‐well plates (100 μl/well) for coating overnight at 4°C. On the 2nd day, the 96‐well plates were washed three times with PBS containing 0.05% Tween (PBST) and incubated at 37°C for 1 h with 200 μl of blocking solution (PBST solution containing 2% bovine serum albumin). Subsequently, the 96‐well plates were washed three times and incubated for 2 h with 100 μl of diluted sera samples (dilution ratio: 1000). Then, the 96‐well plates were washed with PBST three times and incubated at 37°C for 1 h with the horseradish peroxidase‐conjugated goat anti‐mouse IgG antibody solution (100 μl/well). Subsequently, the 96‐well plates were washed with PBST four times, and incubated in dark for 15 min with the 3,3′,5,5′‐tetramethylbenzidine substrate solution (100 μl/well). Finally, the 96‐well plates were incubated with H_2_SO_4_ solution (100 μl/well) to stop the enzymatic reaction. Then, the optical absorbances (ODs) of the wells were read at 450 nm by the microplate reader.

### 2.16 Measurement of splenocytes proliferation

The obtained splenocytes were seeded in 96‐well plates (6 parallel wells/mouse, 5 × 10^4^ cells/well). Among them, three parallel wells were restimulated for 72 h with the OVA solution (final concentration: 25 μg/ml) and the other three parallel wells without OVA solution were used as negative controls. Then, all cells were incubated for 4 h at 37°C with the CCK‐8 reagent (20 μl/well) and measured with the microplate reader. The splenocyte proliferation index (PI) was calculated with the formula:

$$\mathrm{PI}=\begin{array}{l} \left[\mathrm{OD}\text{(restimulated wells)}-\mathrm{OD}\text{(background wells)}\right]/[\mathrm{OD}\left(\text{non}‐\text{restimulated wells}\right)\\ \;-\;\mathrm{OD}\text{(background wells)}]. \end{array}$$

### 2.17 Detection of the cytokines secreted by the splenocytes

The obtained splenocytes were seeded in 12‐well plates (5 × 10^5^ cells/well) and re‐stimulated for 60 h with OVA solution (final concentration: 25 μg/ml). Subsequently, the secretion levels of cytokines IFN‐γ, IL‐6, IL‐4, and TNF‐α in supernatants were detected with ELISA kits as detailed in the manufacturer's instructions and the cells were dyed with the fluorescent dye‐labeled antibodies solutions (anti‐CD3‐APC, anti‐CD8‐PerCP‐Cy5.5, and anti‐CD4‐FITC) to detect the percentage of CD8^+^/CD4^+^ T cells.

### 2.18 Detection of immune memory T cells

The obtained splenocytes (1 × 10^6^ cells/mouse) were stained with fluorescent dye‐labeled antibodies solutions (anti‐CD62L‐APC, anti‐CD44‐PE, anti‐CD8a‐PerCP‐Cy5.5, and anti‐CD4‐FITC) and then detected the percentages of CD44^hi^ CD62L^low^ cells in CD8^+^ T cells and CD4^+^ T cells with the flow cytometer.

### 2.19 Evaluation of histocompatibility of the DC vaccines

The five main tissues (heart, liver, spleen, lung, and kidney) of the mice were collected and fixed with 4% paraformaldehyde solution to prepare the tissue slices. Subsequently, those tissue slices were stained with hematoxylin–eosin staining solution and observed with the optical microscope.

### 2.20 Statistical analysis

The obtained data were statistically analyzed using GraphPad Prism 5 software and the differences between the groups were analyzed using one‐way ANOVA test. The data were expressed as the mean ± standard deviation (Mean ± *SEM*). *p < 0.05, **p < 0.01, and ***p < 0.001 were used to indicate the significant differences.

## 3 RESULTS AND DISCUSSION

### 3.1 Rational design and characterization of Fe_3_O_4_ @Ca/MnCO_3_ nanoparticles

**FIGURE 1:**
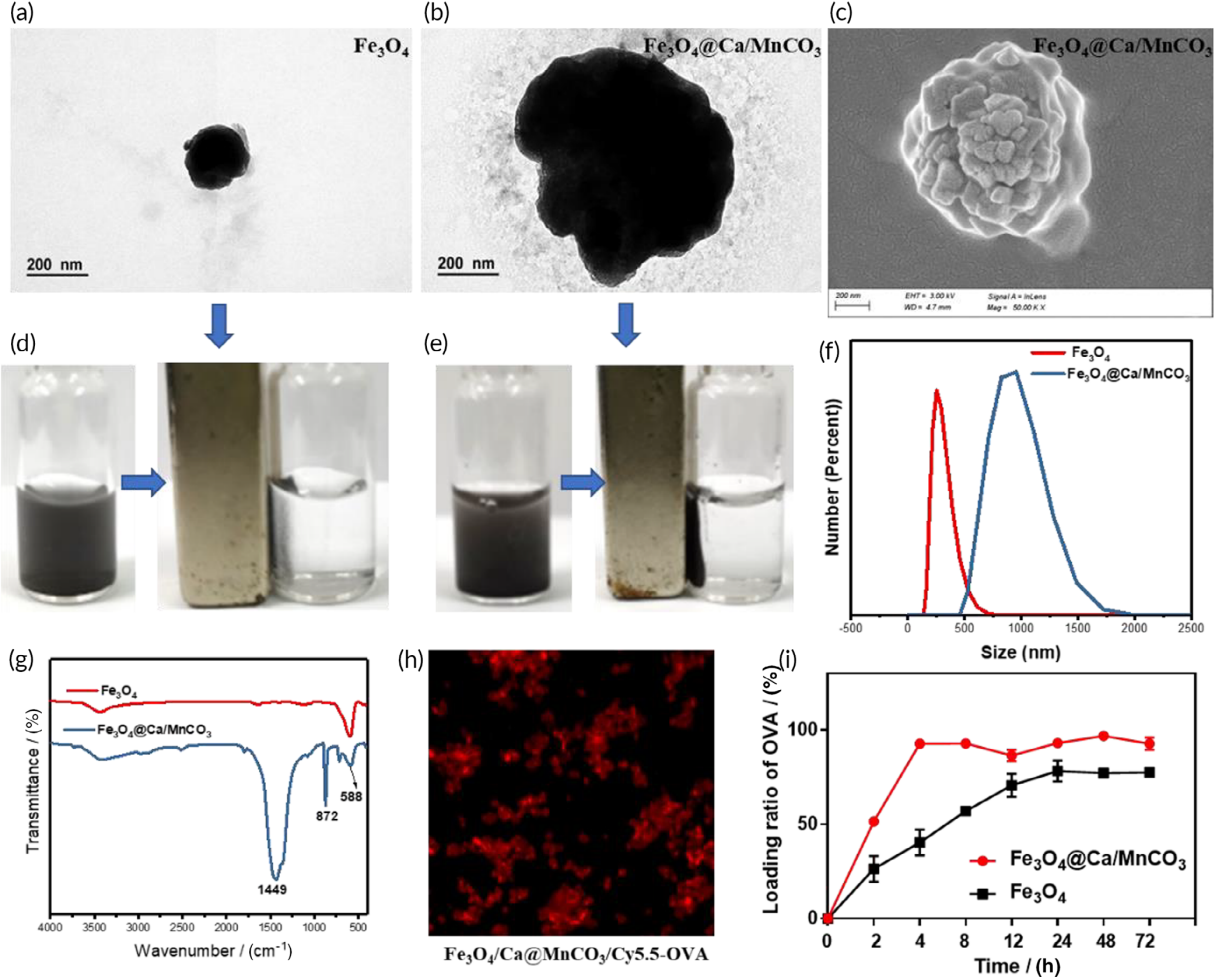
Characterization of Fe_3_O_4_ and Fe_3_O_4_@Ca/MnCO_3_ nanoparticles. Transmission electron microscope images of Fe_3_O_4_ (a) and Fe_3_O_4_@Ca/MnCO_3_ nanoparticles (b). (c) Scanning electron microscope image of Fe_3_O_4_@Ca/MnCO_3_ nanoparticles. The magnetic response capacity of Fe_3_O_4_ (d) and Fe_3_O_4_@Ca/MnCO_3_ nanoparticles (e). (f) The size of Fe_3_O_4_ and Fe_3_O_4_@Ca/MnCO_3_ nanoparticles. (g) FT‐IR spectra of Fe_3_O_4_ and Fe_3_O_4_@Ca/MnCO_3_ nanoparticles. (h) The confocal laser scanning microscope images of Fe_3_O_4_@Ca/MnCO_3_ nanoparticles loaded with Cy5.5‐OVA. (i) the loading ratios of OVA by Fe_3_O_4_ and Fe_3_O_4_@Ca/MnCO_3_ nanoparticles

Multifunctional Fe_3_O_4_@Ca/MnCO_3_ magnetic nanoparticles were synthesized via one‐step method by forming Ca doped MnCO_3_ (Ca/MnCO_3_) coating around Fe_3_O_4_ nanoparticles cores. The size and shape of the Fe_3_O_4_@Ca/MnCO_3_ nanoparticles were investigated by SEM and TEM. The TEM images showed that the size of the Fe_3_O_4_ nanoparticles and Fe_3_O_4_@Ca/MnCO_3_ nanoparticles was ~200 nm (Figure 1a) and ~900 nm (Figure 1b), respectively. In addition, the TEM image of Fe_3_O_4_@Ca/MnCO_3_ nanoparticles taken at low magnification was shown in Figure S1A, Fe_3_O_4_@Ca/MnCO_3_ nanoparticles had uniform particle size and good dispersibility. The SEM image shows that the morphology of the Fe_3_O_4_@Ca/MnCO_3_ nanoparticles is flower‐like (Figures 1c). As the strong magnetic properties of Fe_3_O_4_ nanoparticles (Figure 1d), the obtained Fe_3_O_4_@Ca/MnCO_3_ nanoparticles still kept good magnetic property (Figure 1e). The sizes and zeta potentials of the Fe_3_O_4_ nanoparticles were 255.0 nm and −7.02 mV respectively, and those of Fe_3_O_4_@Ca/MnCO_3_ nanoparticles were 955.4 nm and − 15.7 mV, respectively (Figures 1f), which indicates that Ca/MnCO_3_ coating increased the size of Fe_3_O_4_ nanoparticles and decreased the zeta potential of Fe_3_O_4_ nanoparticles. This will help to reduce the aggregation of magnetic nanoparticles. Meanwhile, we measured size of Fe_3_O_4_@Ca/MnCO_3_ nanoparticles dispersed in deionized water at different times. As shown in Figure S2A,B, the measured sizes of Fe_3_O_4_ nanoparticles increased obviously within 12 h, while the measured sizes of Fe_3_O_4_@Ca/MnCO_3_ nanoparticles remained almost unchanged within 12 h, indicating that the Fe_3_O_4_@Ca/MnCO_3_ nanoparticles maintain good dispersion over 12 h, which will not affect their application in DCs.

Subsequently, the FT‐IR and XRD were used to analyze the crystal structure of the Fe_3_O_4_@Ca/MnCO_3_ nanoparticles. The FT‐IR spectra of Fe_3_O_4_ and Fe_3_O_4_@Ca/MnCO_3_ nanoparticles are shown in Figure 1g, the absorption peak at around 588 cm^−1^ was the characteristic absorption of Fe–O–Fe stretching vibration,^37^ while the absorption band at 1449 and 872 cm^−1^ in the spectrum corresponds to the stretching and bending vibrations of CO_3_^2−^ ^38^ respectively. The XRD spectra of Fe_3_O_4_ and Fe_3_O_4_@Ca/MnCO_3_ nanoparticles are shown in Figure S1B. The diffraction peaks around 2*θ* = 23.45°, 30.15°, 36.47°, 39.98°, and 49.42° corresponded to the (012), (104), (110), (113), and (116) crystal planes of standard XRD pattern of MnCO_3_ (JCPDS no. 44‐1472), respectively. The diffraction peaks around 2*θ* = 18.28°, 35.48°, 43.06°, 56.98°, and 62.66° corresponded to the (111), (311), (400), (511), and (440) crystal planes of standard XRD pattern of Fe_3_O_4_ nanoparticles (JCPDS no. 65‐3107),^39^ respectively. The XRD pattern of Fe_3_O_4_@Ca/MnCO_3_ does not contain the crystal form of CaCO_3_, indicating that Ca does not exist in CaCO_3_ form in the crystal. While the inductively coupled plasma (ICP) elemental analysis result showed that the Fe_3_O_4_@Ca/MnCO_3_ nanoparticles contained Fe (14.1%, w/w), Ca (14.8%, w/w), and Mn (11.3%, w/w) element. These results indicate that Ca/MnCO_3_ was successfully coated around the surface of the Fe_3_O_4_ nanoparticles, and Fe_3_O_4_@Ca/MnCO_3_ nanoparticles were successfully synthesized.

In addition, the specific surface area results of Fe_3_O_4_ and Fe_3_O_4_@Ca/MnCO_3_ nanoparticles are shown in Figure S1C,D. The specific surface area of Fe_3_O_4_ nanoparticles was extremely low (7.345 m^2^/g), while the specific surface area of Fe_3_O_4_@Ca/MnCO_3_ nanoparticles increased to 57.67 m^2^/g. Thus, the coating of Ca/MnCO_3_ increased the specific surface area, which will help to improve the loading level of OVA. The magnetization curves of Fe_3_O_4_ and Fe_3_O_4_@Ca/MnCO_3_ nanoparticles are shown in Figure S2C. The magnetic property (Ms) of Fe_3_O_4_ nanoparticles was 80.1 emu/g, and that of Fe_3_O_4_@Ca/MnCO_3_ nanoparticles was 13.36 emu/g. Although the coating of Ca/MnCO_3_ reduces the Ms of Fe_3_O_4_, this level of Ms (13.36 emu/g) is sufficient for biological applications (usually 7–22 emu/g).^40^, ^41^ Moreover, no hysteresis curve was observed, which indicates the characteristic superparamagnetic behavior of the Fe_3_O_4_ and Fe_3_O_4_@Ca/MnCO_3_ nanoparticles.^42^

### 3.2 Antigen loading and release capacity of Fe_3_O_4_ @Ca/MnCO_3_ nanoparticles

The tumor model antigen, OVA, was selected to test the antigen loading capacity of Fe_3_O_4_@Ca/MnCO_3_ nanoparticles. After adsorbing the fluorescently labeled OVA (Cy5.5‐OVA), the resulting Fe_3_O_4_@Ca/MnCO_3_/Cy5.5‐OVA complexes emitted red fluorescence (Figure 1h). Subsequently, the OVA loading capabilities by Fe_3_O_4_ and Fe_3_O_4_@Ca/MnCO_3_ nanoparticles were measured. As shown in Figure 1i, the OVA loading ability of the Fe_3_O_4_ nanoparticles was low, 5 mg of Fe_3_O_4_ nanoparticles only loaded 120 μg OVA within 4 h, while 5 mg of Fe_3_O_4_@Ca/MnCO_3_ nanoparticles could load 280 μg OVA within 4 h and remained intact within 72 h. Those results prove that Ca/MnCO_3_ coating increase the antigen loading capacity of Fe_3_O_4_ nanoparticles, which may be attributed to the fact that the coating of Ca/MnCO_3_ increased the specific surface area that provides more adsorption sites. In addition, several literatures have reported that the amounts of OVA loaded on carriers is usually in the range of 7.32–220 μg/mg.^43^, ^44^, ^45^, ^46^, ^47^, ^48^, ^49^ In this study, the OVA amount loaded on Fe_3_O_4_@Ca/MnCO_3_ nanoparticles was 56 μg/mg, which has met the application of immunization.

Subsequently, the release of OVA was detected with BCA kit at different times, as shown in Figure S1D. OVA was quickly released from the Fe_3_O_4_@Ca/MnCO_3_/OVA nanoparticles at acidic buffer solution and reached the maximum release ratio within 1 h.

### 3.3 Nanoparticles enhance the maturation of BMDCs and antigen presentation

Exogenous antigens with low immunogenicity and stability,^50^ are usually presented to CD4^+^ T cells through the MHC class II molecules. However, nanoparticles act as reservoirs of antigens, can promote antigen uptake, and present antigen to CD8^+^ T cells with MHC class I molecules to induce cellular immunity.^51^, ^52^ In addition, costimulatory molecules such as CD40, CD80, and CD86 are necessary for all antigen presentations.

**FIGURE 2:**
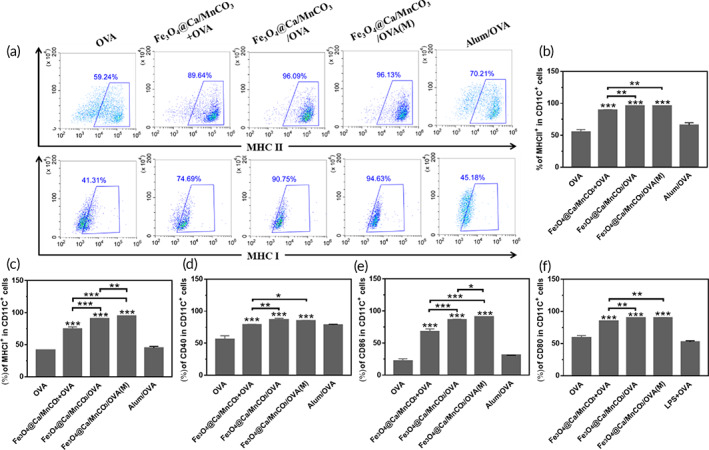
Maturation of bone marrow‐derived dendritic cells and antigen presentation induced by Fe_3_O_4_@Ca/MnCO_3_ nanoparticles. (a) Representative scatter plots of MHC II and MHC I, and the corresponding percentages of MHC II (b), MHC I (c), CD40 (d), CD86 (e), and CD80 (f) molecules expressed on CD11c^+^ DC cells. *p < 0.05, **p < 0.01, and ***p < 0.001

In this study, the isolated BMDCs were treated with the magnetic Fe_3_O_4_@Ca/MnCO_3_ nanoparticles and other controls for 24 h, and their expressions of OVA‐specific MHC I, MHC II, CD40, CD80, and CD86 molecules were analyzed. The results are shown in Figure 2a–f. The Fe_3_O_4_@Ca/MnCO_3_ + OVA groups (OVA was simply mixed with Fe_3_O_4_@Ca/MnCO_3_ nanoparticles) showed increased level of MHC and costimulatory molecules. However, the levels of MHC and costimulatory molecules in Fe_3_O_4_@Ca/MnCO_3_/OVA group (OVA was loaded by Fe_3_O_4_@Ca/MnCO_3_ nanoparticles) were significantly higher. It is worth noting that, application of magnetic fields can significantly increase the expression of OVA‐specific MHC I and CD86 molecules of Fe_3_O_4_@Ca/MnCO_3_/OVA group, the results indicate that the load of antigen on Fe_3_O_4_@Ca/MnCO_3_ nanoparticles and application of magnetic fields could promote antigen cross‐presentation, and further promote the proliferation of CD8^+^ T cells. To explore the mechanisms of magnetic nanoparticles to activate DCs, more data about their interaction with DCs were necessary.

In addition, the levels of BMDC maturation and antigen presentation induced by Fe_3_O_4_/OVA and Fe_3_O_4_@Ca/MnCO_3_/OVA group were compared. The OVA loading amounts of Fe_3_O_4_ and Fe_3_O_4_@Ca/MnCO_3_ nanoparticles were 24 and 56 μg/mg, respectively. In order to ensure consistent OVA loading amount on Fe_3_O_4_ and Fe_3_O_4_@Ca/MnCO_3_ nanoparticles, we added 120 μg OVA into 5 mg of Fe_3_O_4_ or Fe_3_O_4_@Ca/MnCO_3_ suspension. Subsequently, Fe_3_O_4_ or Fe_3_O_4_@Ca/MnCO_3_ nanoparticles loaded with OVA were co‐cultured with BMDCs. The results are shown in Figure S3. The Fe_3_O_4_/OVA groups showed low level of expressions of MHC and costimulatory molecules. By contrast, the levels of MHC and costimulatory molecules in Fe_3_O_4_@Ca/MnCO_3_/OVA group were significantly higher. These results indicate that Ca/MnCO_3_ shell of Fe_3_O_4_@Ca/MnCO_3_/OVA group can significantly enhance antigen presentation and stimulate DC maturation, which will promote subsequent immune response.

### 3.4 Cellular uptake of the magnetic nanoparticles

**FIGURE 3:**
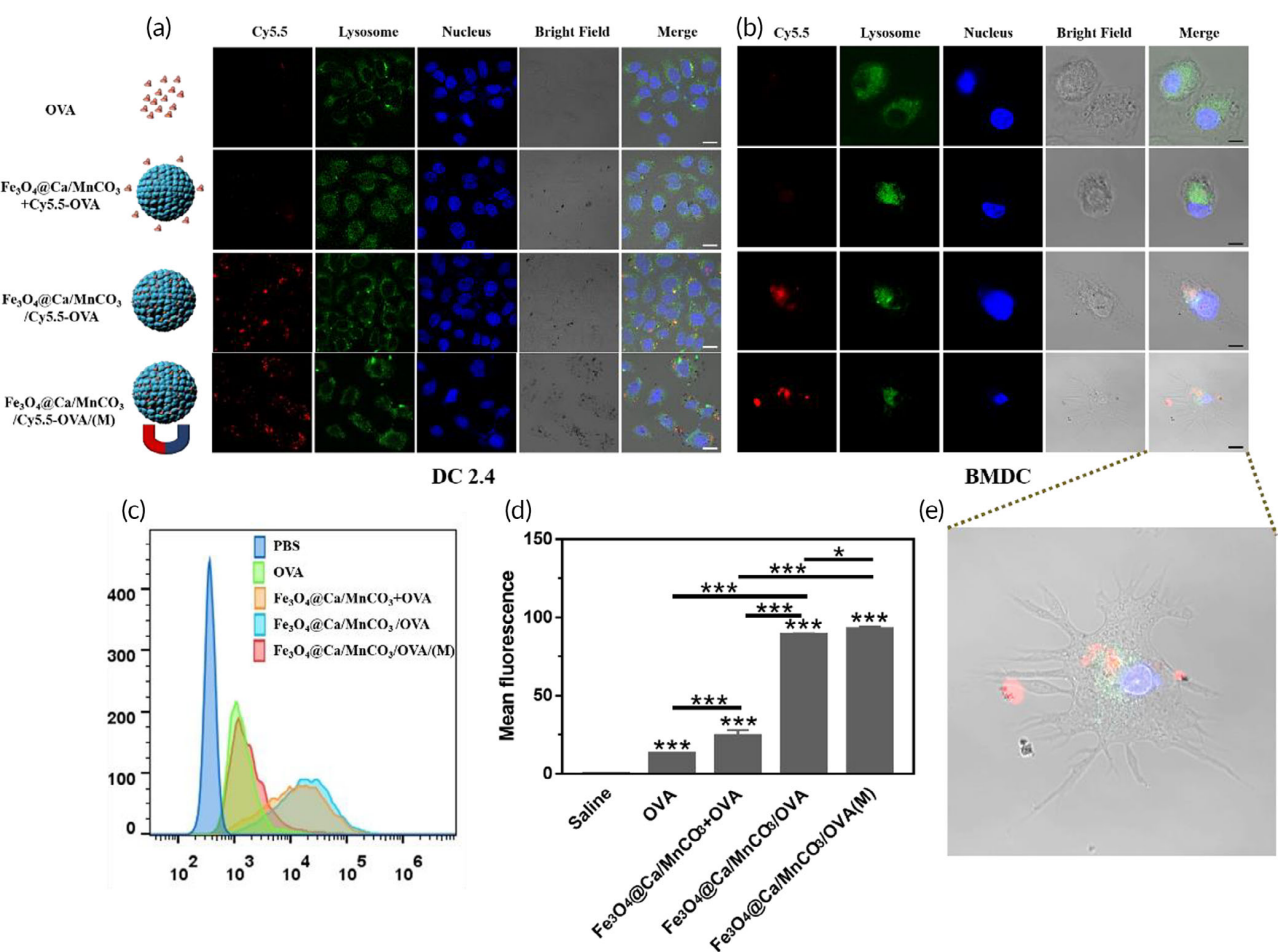
Uptake of antigen by DCs. Confocal laser scanning microscope images of DC2.4 (a) and bone marrow‐derived dendritic cells (BMDCs) (b) after incubation with vaccine formulations for 6 h, OVA labeled with Cy5.5 (red fluorescence), lysosome labeled with FITC (green fluorescence), and nucleus labeled with DAPI (blue fluorescence). White scar bar: 20 μm, Black scar bar: 10 μm. (c) The flow histograms of internalized Cy5.5‐OVA in each group, and (d) the corresponding statistical analysis. (e) The enlarged images of BMDC in Fe_3_O_4_@Ca/MnCO_3_/OVA(M). *p < 0.05, **p < 0.01, and ***p < 0.001

Antigen uptakes are key steps for APCs' activation in the generation of potent immune responses.^53^ The antigen delivery performance of Fe_3_O_4_@Ca/MnCO_3_ nanoparticles to DC2.4 and BMDCs was examined. As the CLSM (Figure 3a,b) images show, in comparison with the Cy5.5‐OVA alone and Fe_3_O_4_@Ca/MnCO_3_ + Cy5.5‐OVA groups, the Fe_3_O_4_@Ca/MnCO_3_/Cy5.5‐OVA containing groups significantly increased the antigen internalization into the lysosome, revealing that Fe_3_O_4_@Ca/MnCO_3_/OVA nanoparticles can deliver OVA to DCs more effectively after loading OVA. Moreover, the flow cytometry was used to evaluate whether Fe_3_O_4_@Ca/MnCO_3_/Cy5.5‐OVA nanoparticles can further enhance the delivery of OVA under magnetic field. As shown in Figure 3c,d, the Fe_3_O_4_@Ca/MnCO_3_ nanoparticles under magnetic field significantly increased the antigen internalization, which may be due to the fact that the magnetic field actively and rapidly pulls all magnetic particles into contact with cells. Surprisingly, the BMDC in Fe_3_O_4_@Ca/MnCO_3_/OVA group presented dendritic morphology (Figure 3e), which indicates that BMDCs were completely activated.

### 3.5 Intracellular degradation of the Fe_3_O_4_ @Ca/MnCO_3_ nanoparticles

**FIGURE 4:**
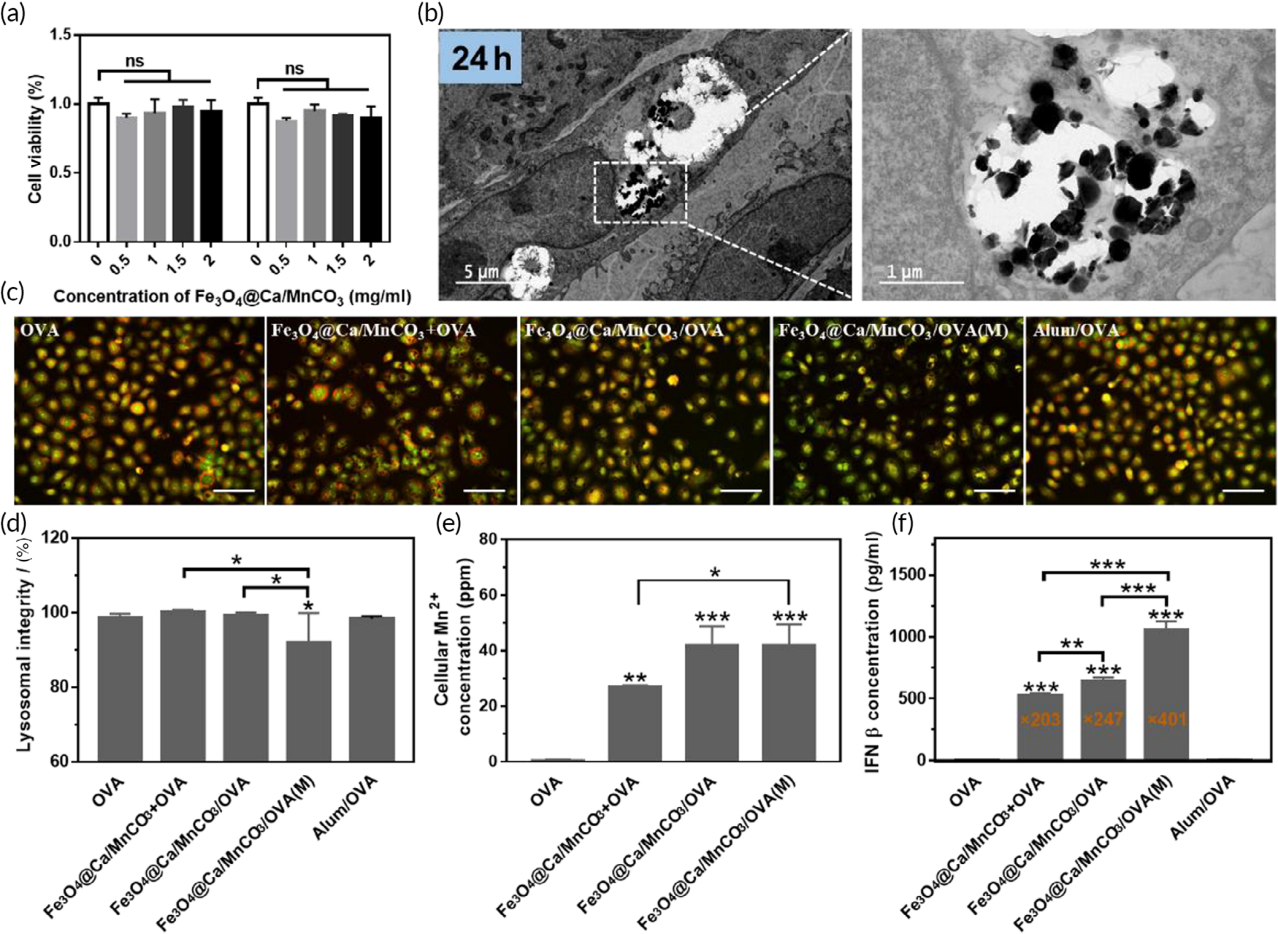
The degradation of Fe_3_O_4_@Ca/MnCO_3_ nanoparticles in the DCs. (a) The toxicity of the Fe_3_O_4_@Ca/MnCO_3_ nanoparticles to DC2.4 in the presence or absence of a magnetic field. (b) TEM observation of DC2.4 treated with Fe_3_O_4_@Ca/MnCO_3_ nanoparticles for 24 h. (c) Lysosomal integrity observation of DC2.4 exposed to vaccine preparations for 24 h, and (d) quantitative results of lysosomal integrity. Scar bar: 50 μm. (e) Intracellular Mn^2+^ level in DC2.4 after incubation with vaccine preparations for 24 h, and (f) the IFN‐β level in DCs after incubation with vaccine preparations for 24 h. *p < 0.05, **p < 0.01, and ***p < 0.001

The degradation of vaccine carriers with good biological safety in cytoplasm is critical to release antigens and adjuvants for subsequent antigen presentation. Thus, it is necessary to observe degradation of Fe_3_O_4_@Ca/MnCO_3_ nanoparticles in the cytoplasm of DCs. First, the toxicities of Fe_3_O_4_@Ca/MnCO_3_ nanoparticles to DCs under the presence or absence of the magnetic field were tested, as shown in Figure 4a, there is no obvious cytotoxicity when at the concentrations of Fe_3_O_4_@Ca/MnCO_3_ nanoparticles <2 mg/ml, indicating that Fe_3_O_4_@Ca/MnCO_3_ nanoparticles under presence or absence of a magnetic field had good safety. Subsequently, the morphology of Fe_3_O_4_@Ca/MnCO_3_ nanoparticles in DCs was observed with TEM, and the result is shown in Figure 4b. The Fe_3_O_4_@Ca/MnCO_3_ nanoparticles in DC2.4 were broken at 24 h. This suggests that the Fe_3_O_4_@Ca/MnCO_3_ nanoparticles dissolved in the weak acid environment of the lysosomes. Then, acridine orange dye was used to detect the lysosome integrity. Results (Figure 4c,d) show that the magnetic field will accelerate the rupture of lysosomes, which can promote the release of antigens into the cytoplasm.

In addition, intracellular Ca^2+^ and Mn^2+^ levels were determined to further confirm the degradation of the Fe_3_O_4_@Ca/MnCO_3_ nanoparticles in the lysosomes. As shown in Figure 4e, the intracellular Mn^2+^ level in the OVA alone group was very low, while the intracellular Mn^2+^ levels were significantly increased in the Fe_3_O_4_@Ca/MnCO_3_‐containing groups, further proving the degradation of Fe_3_O_4_@Ca/MnCO_3_ nanoparticles in lysosomes. Moreover, the intracellular Mn^2+^ can stimulate the production of the type I‐IFN,^23^ and thereby increasing the effectiveness of the CD8^+^ T cell response.^54^ IFN‐β is one kind of type I IFN and an important indicator of the STING pathway activation. We investigated whether Mn^2+^ produced by the degradation of intracellular Fe_3_O_4_@Ca/MnCO_3_ nanoparticles could activate the STING pathway in DCs. As shown in Figure 4f, the concentration of IFN‐β in Fe_3_O_4_@Ca/MnCO_3_‐containing groups was significantly higher than OVA alone groups. Among them, Fe_3_O_4_@Ca/MnCO_3_/OVA under magnetic field can significantly increase concentration of IFN‐β. The results indicate that Fe_3_O_4_@Ca/MnCO_3_ nanoparticles and magnetic field can activate the STING pathway. Overall, the concentration of IFN‐β in Fe_3_O_4_@Ca/MnCO_3_/OVA(M) group was the highest, which may be the reason for the highest expression of MHC I and CD86 in this group.

**FIGURE 5:**
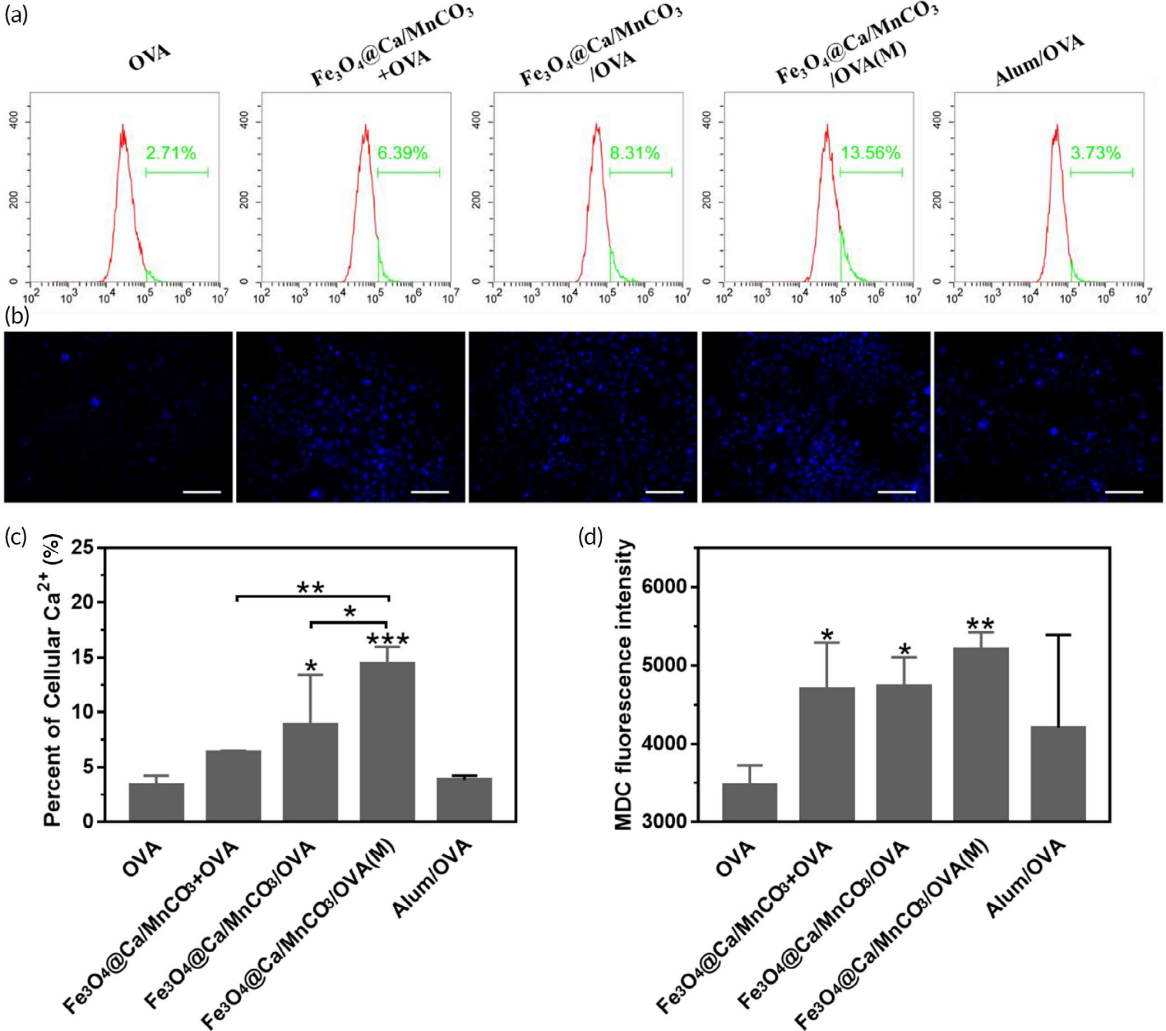
(a) Intracellular Ca^2+^ level and (c) quantitative results. (b) monodansylcadaverine staining images in DC2.4 after incubation with vaccine preparations for 24 h, and (d) the corresponding fluorescence intensity of MDC. Scar bar: 50 μm. *p < 0.05, **p < 0.01, and ***p < 0.001

Subsequently, the intracellular Ca^2+^ was detected with the fluorescent dye Fluo‐4/AM.^55^ As shown in Figure 5a,c, the intracellular Ca^2+^ level in the OVA alone and Alum/OVA groups was low, while the intracellular Ca^2+^ level was significantly increased in the Fe_3_O_4_@Ca/MnCO_3_‐containing groups, further proving the degradation of Fe_3_O_4_@Ca/MnCO_3_ in cytoplasm. Among them, the application of magnetic field lead to the highest level of intracellular Ca^2+^, which might be attributed to the largest amount of their internalization in lysosomes (Figure 3b,c). It is worth mentioning that, intracellular Ca^2+^ plays an important role in the induction or activation of autophagy,^25^ which will be beneficial to antigen cross‐presentation.^56^ In this study, MDCs staining was used to label the autophagic vacuoles of DCs induced by Fe_3_O_4_@Ca/MnCO_3_ nanoparticles, and the results are shown in Figure 5b,d. The OVA alone and Alum/OVA groups only had weak blue fluorescent, while strong blue fluorescence intensity (autophagic vacuoles) appeared in Fe_3_O_4_@Ca/MnCO_3_‐containing groups, indicating that the addition of Fe_3_O_4_@Ca/MnCO_3_ nanoparticles promoted the production of autophagosome. STING signaling pathway (by Mn^2+^) and autophagy (by Ca^2+^) can promote antigen cross‐presentation, which helps to produce cytotoxic T lymphocytes. Overall, the Fe_3_O_4_@Ca/MnCO_3_/OVA(M) group had the highest intracellular Ca^2+^ and Mn^2+^ levels, which may be attributed to the application of magnetic field increasing the amounts of magnetic nanoparticles entering DC.

### 3.6 Magnetic nanoparticles activated DC promote the proliferation and activation of CD8 ^+^ T lymphocytes in vitro

**FIGURE 6:**
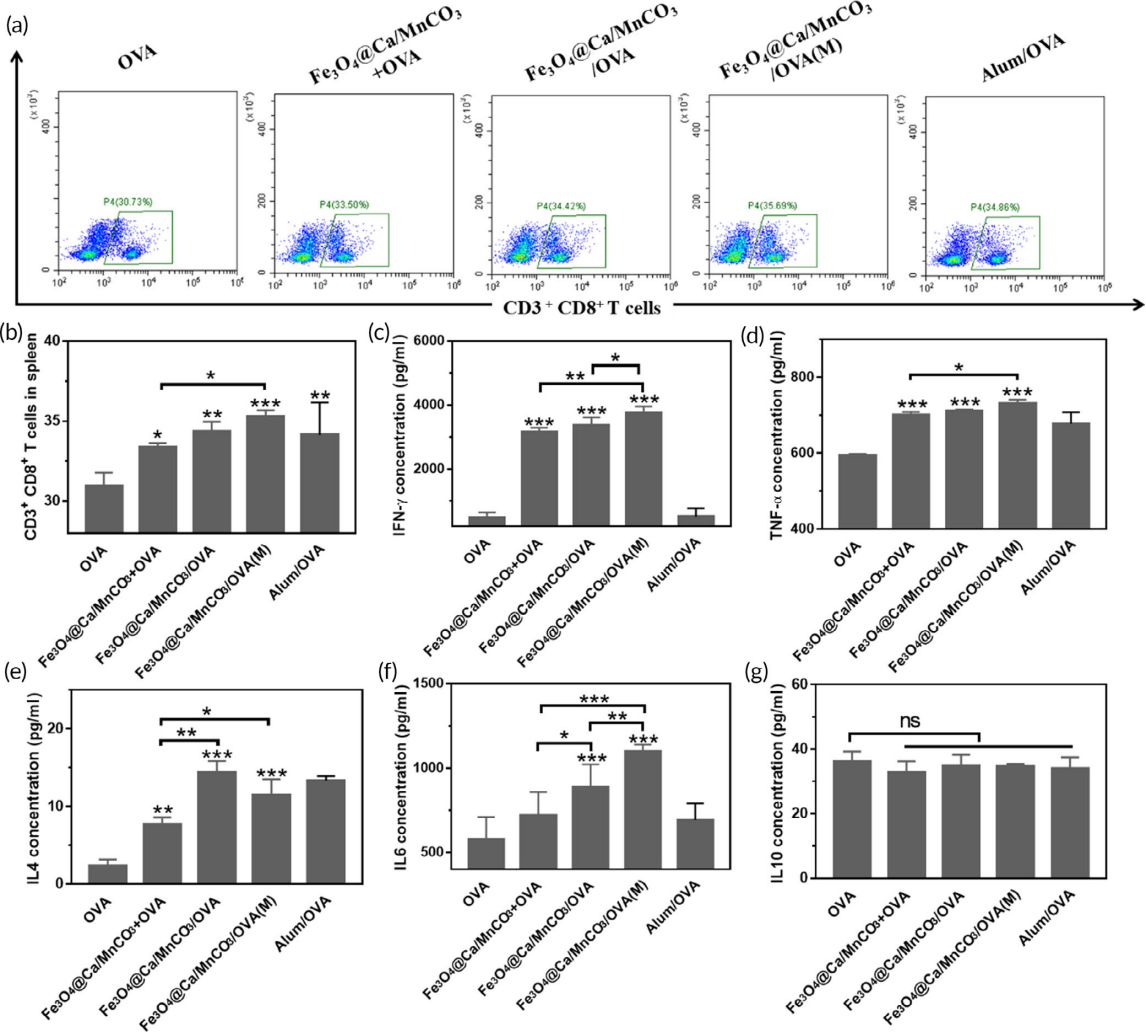
Bone marrow‐derived dendritic cells loaded with magnetic nanoparticles were co‐cultured with naive splenocytes for 48 h. (a, b) the ratio of CD8^+^ T cells in T cells, and the level of IFN‐γ (c), TNF‐α (d), IL‐4 (e), IL‐6 (f), and IL‐10 (g) secreted by T cells. *p < 0.05, **p < 0.01, and ***p < 0.001

In view of the fact that magnetic nanoparticles could enhance antigen uptake, promote DCs activation, and enhance antigen cross‐presentation under a magnetic field, the Fe_3_O_4_@Ca/MnCO_3_ nanoparticles activated BMDCs were then incubated with naive splenocytes, and evaluated its function to stimulate CD8^+^ T cell proliferation and cytokine secretion in vitro. As shown in Figure 6a,b, The DCs activated by the Fe_3_O_4_@Ca/MnCO_3_ containing nanoparticles could significantly increase the proportion of CD8^+^ T cells in T cells, which will contribute to cellular immunity.

Subsequently, the supernatant of the co‐culture of BMDCs and splenocytes was tested. IFN‐γ is an important cytokine to promote the production of antibody IgG2a and differentiation of CD8^+^ T cells into cytotoxic T lymphocyte (CTLs).^57^ And TNF‐α is also highly related to anti‐tumor immunity.^58^, ^59^ IL‐6 can participate in the regulation of cellular and humoral immune responses.^60^, ^61^ IL‐4 can regulate the production of antibody IgG1 and promote humoral immunity.^62^ IL‐10 is a master regulator of immunity to balance immune responses.^63^ As shown in Figure 6c–f, the DCs activated by the magnetic nanoparticles would significantly activate T cells, express IFN‐γ, TNF‐α, and IL‐4. However, in comparison with Fe_3_O_4_@Ca/MnCO_3_ + OVA activated DCs, the Fe_3_O_4_@Ca/MnCO_3_/OVA activated DCs significantly increased the level of IL‐4 and IL‐6. Moreover, in comparison with Fe_3_O_4_@Ca/MnCO_3_/OVA activated DCs without magnetic field, the Fe_3_O_4_@Ca/MnCO_3_/OVA activated DCs with magnetic field significantly increased the level of IFN‐γ and IL‐6, while did not increase the level of IL‐4. In addition, the levels of IL‐10 secretion in each group were similar (Figure 6g), indicating that nanoparticles activated DCs would not trigger immunomodulatory effects to downregulate the expression of these anti‐tumor related cytokines. These results indicate that the magnetic nanoparticles can increase the secretion of cytokines and the application of magnetic field can promote secretion of cytokines that contribute to cellular immunity.

### 3.7 Antigen migration to peripheral immune organs

**FIGURE 7:**
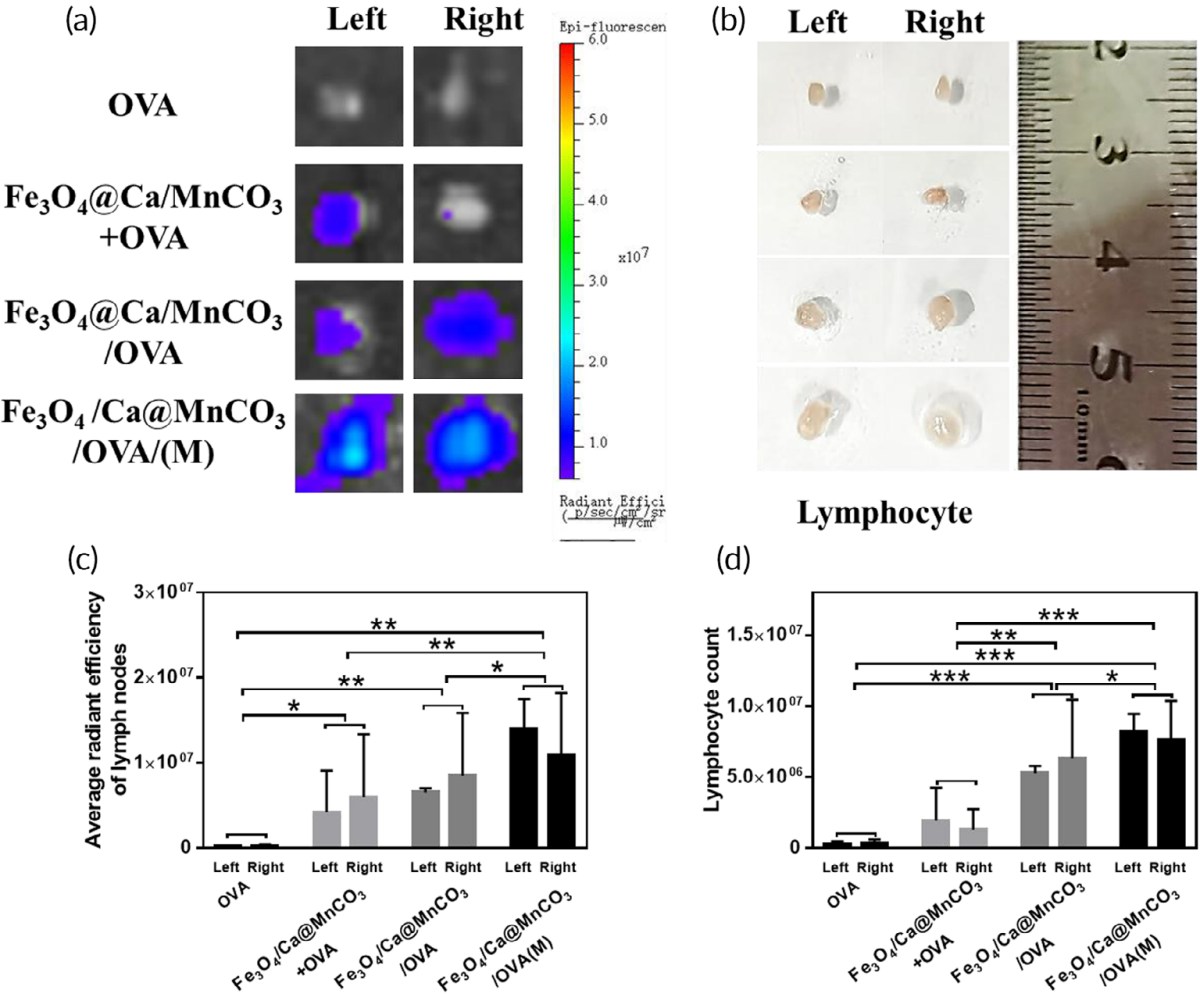
Observation of the migration of antigen taken up by bone marrow‐derived dendritic cell to lymph nodes. (a) Observation of Cy5.5‐OVA at lymph nodes from each group of mice, and (c) the corresponding average radiant efficiency. (b) Observation of sizes of lymph nodes from each group of mice, and (d) the corresponding count of lymph node cells. *p < 0.05, **p < 0.01, and ***p < 0.001

Activated DCs are more likely to migrate from the high endothelial venules of the lymph nodes to the lymph nodes.^64^ Moreover, localization of DCs in the lymph nodes can induce T cell responses faster.^65^ As shown in Figure 7a,c, in comparison with Cy5.5‐OVA‐actived DCs, the fluorescence intensities of lymph nodes in the Fe_3_O_4_@Ca/MnCO_3_/Cy5.5‐OVA activated DCs groups were obviously increased. Among them, the fluorescence intensity in the Fe_3_O_4_@Ca/MnCO_3_/OVA/(M) activated DCs group was the highest. In addition, it can be seen that the antigen‐loaded nanoparticle groups enlarged the sizes of lymph nodes and increased the number of lymphocytes (Figure 7b,d). Among them, Fe_3_O_4_@Ca/MnCO_3_/OVA/(M) activated DCs group had largest lymph nodes. These results indicate that after treatment with Fe_3_O_4_@Ca/MnCO_3_ and magnetic field, DCs can migrate more to lymph nodes and promote the proliferation of T lymphocytes.

**FIGURE 8:**
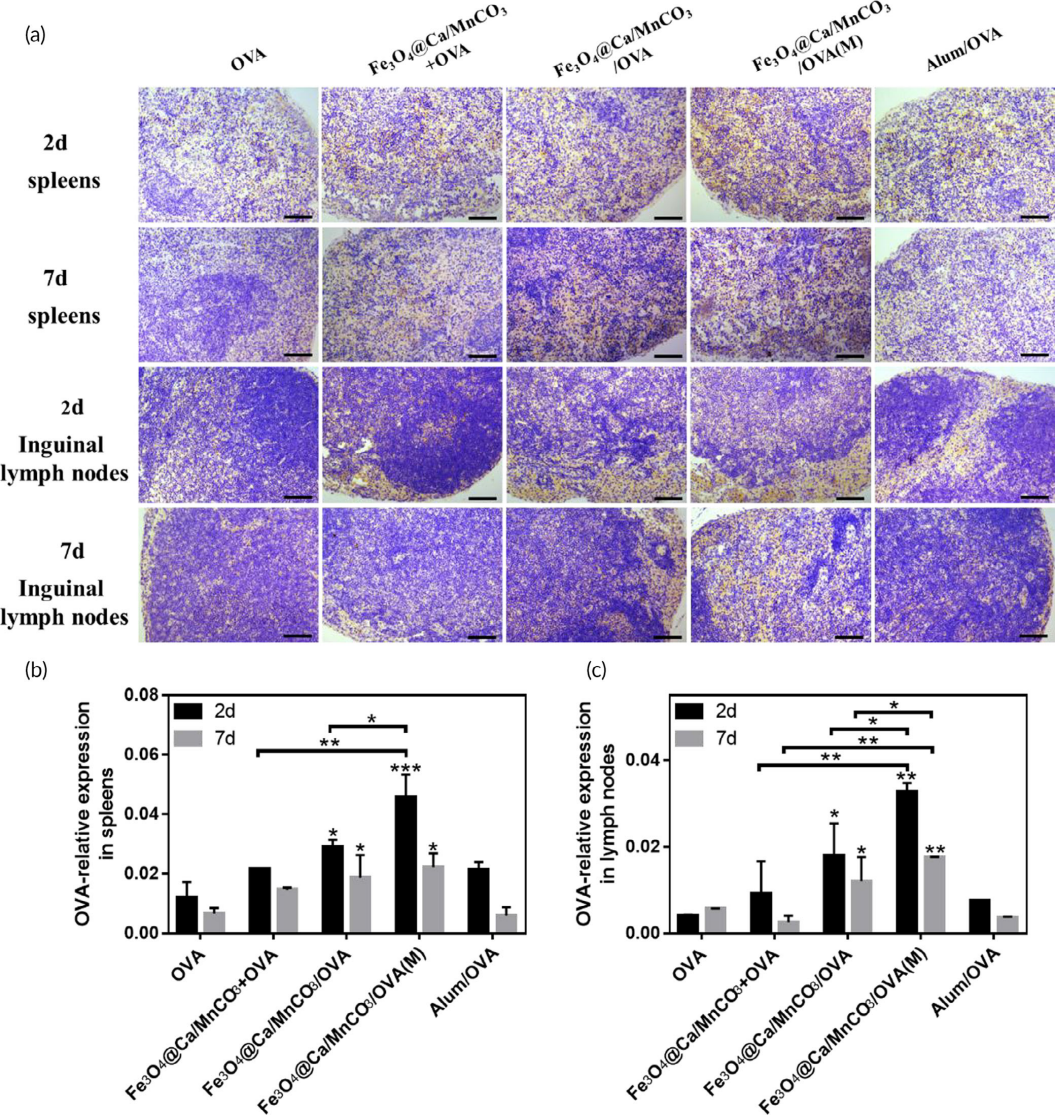
(a) Immunohistochemical images of the spleens and lymph nodes of immunized mice, OVA is represented in yellow areas, scar bar: 100 μm, and the corresponding statistical results of spleens (b) and lymph nodes (c). *p < 0.05, **p < 0.01, and ***p < 0.001

In addition, the immunohistochemical analysis was conducted to explore the ability of activated DCs to reach the peripheral immune organs and present antigen to T lymphocytes. To aim this, the mice were subcutaneously injected with activated DCs and their spleens and lymph nodes were collected for the immunohistochemical analysis on 2nd and 7th day. As shown in Figure 8, the OVA amount in the spleens and lymph nodes of all groups on 2nd day was lower than that of on 7th day. Compared with the OVA alone and Fe_3_O_4_@Ca/MnCO_3_ + OVA activated DCs groups, the OVA amount (brown area) in the Fe_3_O_4_@Ca/MnCO_3_/OVA activated DCs groups obviously increased on 2nd and 7th day. Among them, the OVA amount of the Fe_3_O_4_@Ca/MnCO_3_/OVA(M) activated DCs group was the highest. The above results also indicate that the DC activated by the magnetic nanoparticles and magnetic field facilitated the delivery of antigens and promoted the migration of DCs to peripheral immune organs.

### 3.8 DC vaccine induces an effective immune response in vivo

**FIGURE 9:**
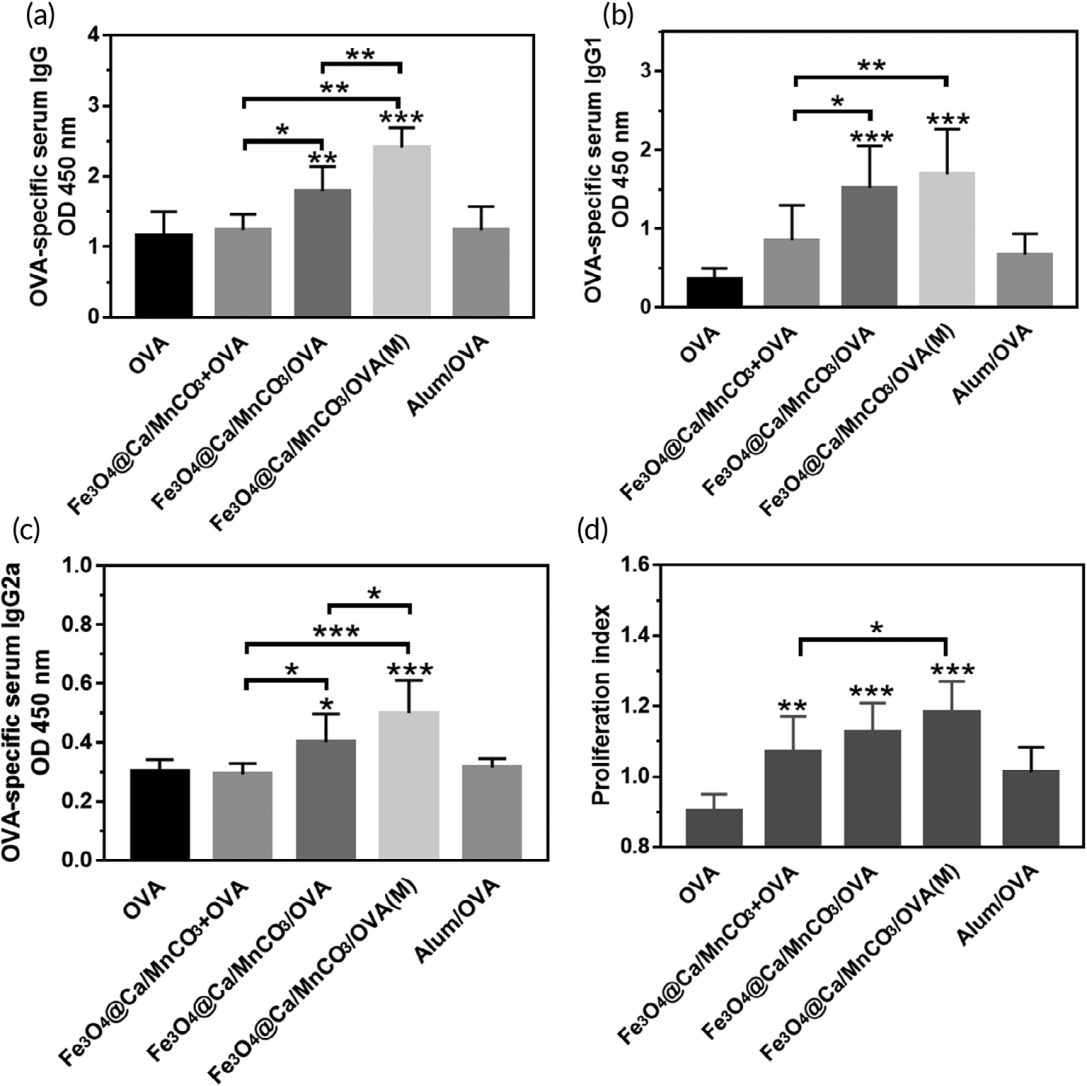
The mice immunized with each dendritic cell vaccine. Serum OVA specific IgG (a), IgG1 (b) and IgG2a (c) titer detected by ELISA. (d) Splenocytes proliferation detected by CCK‐8 kit. *p < 0.05, **p < 0.01, and ***p < 0.001

Antibody titer is a key indicator of the level of humoral and cellular immunity. The OVA‐specific antibody titers in the sera of the immunized mice were measured by ELISA. As shown in Figure 9a–c, in comparison with OVA alone, Alum/OVA, and Fe_3_O_4_@Ca/MnCO_3_ + OVA activated DCs, the antibody titers of the Fe_3_O_4_@Ca/MnCO_3_/OVA activated DCs significantly increased, which indicates that Fe_3_O_4_@Ca/MnCO_3_/OVA activated DCs can enhance the immune response. It is worth noting that, the level of IgG1 and IgG2a induced by Fe_3_O_4_@Ca/MnCO_3_/OVA(M) activated DCs was significantly higher than that of Fe_3_O_4_@Ca/MnCO_3_/OVA activated DCs, while the level of IgG1 of them was basically the same (Figure 9b). These results show that Fe_3_O_4_@Ca/MnCO_3_ loaded with antigen can effectively increase the production of antibodies induced by DC vaccine and the application of magnetic field can further enhance the production of antibodies related to cellular immunity induced by DC vaccine. This may be attributed to the magnetic field promoting the entry of magnetic nanoparticles into DCs and subsequently enhancing the cross presentation of antigens.

Subsequently, the immunological evaluation of Fe_3_O_4_/OVA or Fe_3_O_4_@Ca/MnCO_3_/OVA nanoparticles activated DCs was carried out in vivo. The OVA‐specific antibody titers in the sera of the immunized mice were measured. As shown in Figure S4, in comparison with Fe_3_O_4_/OVA activated DCs, the IgG, IgG1, and IgG2a antibody titers of the Fe_3_O_4_@Ca/MnCO_3_/OVA activated DCs significantly increased, which indicates that Ca/MnCO_3_ shell can enhance the humoral and cellular immune response.

The spleens contain a variety of immune cells, which play important roles in immune responses.^66^ When exposed to the same antigen again, the cells will proliferate rapidly and the T cells will produce fast and effective immune responses.^67^ Subsequently, the splenocytes proliferation of the immunized mice was evaluated to initially assess the level of immune memory. The results are shown in Figure 9d, in comparison with the OVA alone and Alum/OVA activated DCs, the splenocyte PI of the Fe_3_O_4_@Ca/MnCO_3_ containing groups activated DCs increased. Among them, the splenocyte PI of the Fe_3_O_4_@Ca/MnCO_3_/OVA(M) activated DCs was the highest. It demonstrates that DC activated with magnetic nanoparticles and magnetic field can promote the proliferation of splenocytes and causes a stronger immune response to re‐exposed antigens.

**FIGURE 10:**
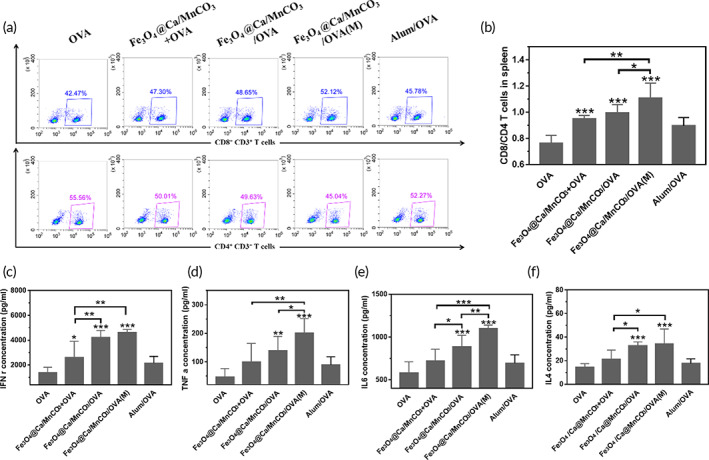
Stimulation and specificity of T cell responses. (a, b) The ratio of CD8^+^/CD4^+^ T cells in T cells, and the level of IFN‐γ (c), TNF‐α (d), IL‐6 (e), and IL‐4 (f) secreted by T cells. *p < 0.05, **p < 0.01, and ***p < 0.001

Then, the proportion of CD8^+^ T cell in splenocytes and the cytokine secretion level was measured, as shown in Figure 10a,b, the DCs activated by magnetic nanoparticles could significantly increase the proportion of CD8^+^ T cells, and Fe_3_O_4_@Ca/MnCO_3_/OVA(M) activated DCs had highest level of CD8^+^ T cells. Meanwhile, the proportion of CD8^+^ T cell in splenocytes and lymphocytes was measured, as shown in Figure S5. Fe_3_O_4_/OVA activated DCs basically did not promote the proliferation of CD8^+^ T cells, while the Fe_3_O_4_@Ca/MnCO_3_/OVA activated DCs could significantly increase the proportion of CD8^+^ T cells. This indicates that the application of Ca/MnCO_3_ shell is more conducive to increase the proportion of CD8^+^ T cells. Subsequently, the supernatant splenocytes were tested, as shown in Figure 10c–f, the DCs activated by Fe_3_O_4_@Ca/MnCO_3_/OVA significantly increased cytokine secretion. Among them, Fe_3_O_4_@Ca/MnCO_3_/OVA(M) activated DCs had highest level of TNF‐α and IL‐6. While the levels of IL‐4 in the Fe_3_O_4_@Ca/MnCO_3_/OVA activated DCs were basically the same as those in the Fe_3_O_4_@Ca/MnCO_3_/OVA(M) activated DCs. This indicates that the application of magnetic field is more conducive for Fe_3_O_4_@Ca/MnCO_3_/OVA activated DCs to induce cellular immune responses. These are consistent with the results of in vitro co‐incubation (Figure 6c–f).

**FIGURE 11:**
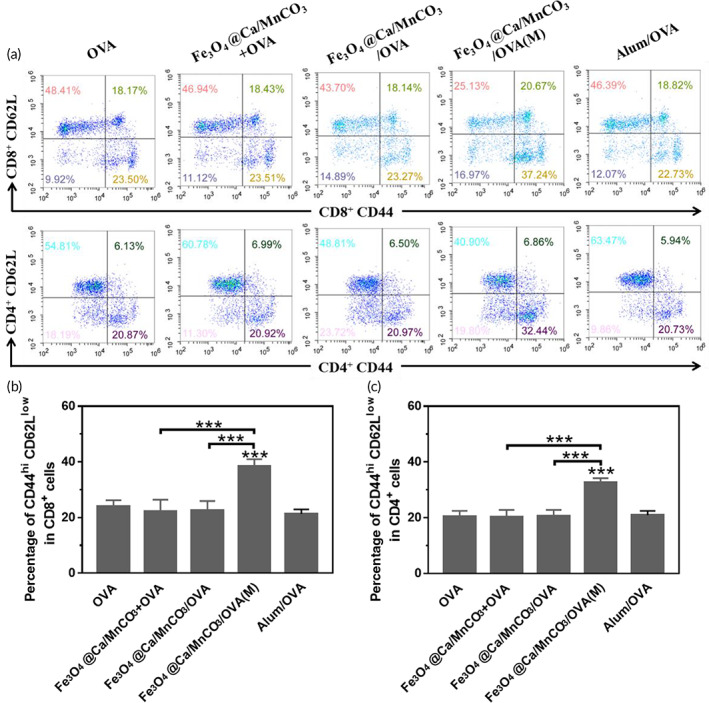
The percentages of CD44^hi^ CD62^low^ cells in CD4^+^ T and CD8^+^ T cells of splenocytes. *p < 0.05, **p < 0.01, and ***p < 0.001

Formation of immune memory T cells is the most important characteristic of vaccination, which plays a key role in immune surveillance by rapidly and potently responding to the re‐exposed antigens.^68^ Memory T cells can be divided into two subgroups, namely, effector memory T cells (T_EM_ cells, CD44^high^ CD62L^low^) and central memory T cells (T_CM_ cells, CD44^high^ CD62L^high^).^69^ Here, the percentage of T_EM_ cells among the splenocytes was detected. As shown in Figure 11, the DCs activated by Fe_3_O_4_@Ca/MnCO_3_/OVA(M) could significantly increase the percentages of CD8^+^ TEM and CD4^+^ T_EM_ cells, which indicates that the application of magnetic field and magnetic nanoparticles can help DCs induce the generation of CD8^+^ T_EM_ and CD4^+^ T_EM_, which can make an immune response instantaneously when antigens invade again.

**FIGURE 12:**
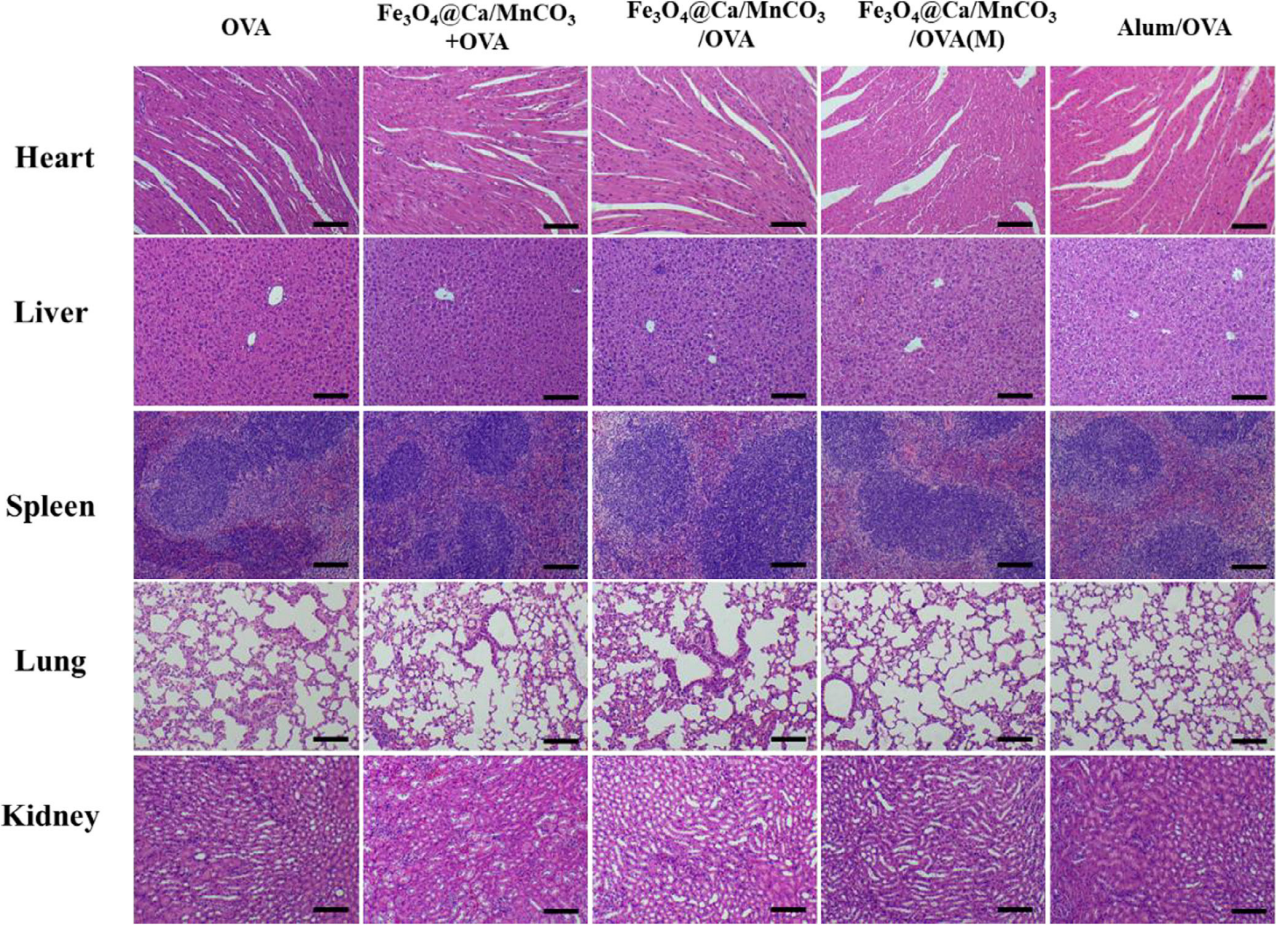
HE staining images of heart, liver, spleen, lung, and kidney sections. Scar bar: 100 μm

Finally, the bio‐safety of the DC vaccine in vivo is critical to their clinical applications. On the 7th day after the last immunization, the heart, liver, spleen, lung, and kidney of the immunized mice were collected to evaluate the histopathological toxicity of the vaccine formulations. As shown in Figure 12, all the organs did not show obvious pathological changes, indicating that DC vaccine had excellent biological safety.

## 4 CONCLUSION

In conclusion, Fe_3_O_4_@Ca/MnCO_3_ magnetic nanoparticles can improve antigen cross presentation and cellular immunity. Due to the fact that the magnetic field actively pulled the magnetic nanoparticles to contact the cells, the Fe_3_O_4_@Ca/MnCO_3_ promoted internalization of antigen. Fe_3_O_4_@Ca/MnCO_3_ nanoparticles in the cell slowly degrade, release Mn^2+^, Ca^2+^ and antigens, increase IFN‐β concentration and autophagy, effectively activate DCs, and promote antigens cross‐presentation. Moreover, DCs activated by magnetic nanoparticles can enter lymph nodes to activate CD8^+^ T cells after immunization, and produce higher levels of antibodies. In comparison with traditional DC vaccine, cytoplasmic antigen delivery with the magnetic nanoparticles provides a new idea for the construction of novel DC vaccines.

## AUTHOR CONTRIBUTIONS

**Linghong Huang**: Methodology; investigation; data curation; formal analysis; writing – original draft. **Zonghua Liu**: Funding acquisition; investigation; writing – review and editing. **Chongjie Wu**: Methodology and investigation. **Jiansheng Lin**: Investigation; conceptualization; writing – review and editing. **Ning Liu**: Writing – original draft; formal analysis; project administration; funding acquisition.

## CONFLICT OF INTEREST

The authors declare that they have no competing financial interests or personal relationships that could have appeared to influence the work reported in this paper.

5

### PEER REVIEW

The peer review history for this article is available at https://publons.com/publon/10.1002/btm2.10400.

## Supporting information


**Appendix S1** Supporting Information.

## Data Availability

The data that support the findings of this study are available from the corresponding author.
