# Supplementary material for: Magnetic nanoparticles enhance the cellular immune response of dendritic cell tumor vaccines by realizing the cytoplasmic delivery of tumor antigens
Source: Bioeng Transl Med. 2022 Sep 9;8(2):e10400. doi: 10.1002/btm2.10400 (PMC10013825; doi:10.1002/btm2.10400)
Supplement: Supplementary file 1 — Appendix S1 Supporting Information. [file BTM2-8-e10400-s001.docx]

**Supporting Information**

**Magnetic nanoparticles enhance the cellular immune response of DC tumor vaccines by realizing the cytoplasmic delivery of tumor antigens**

Linghong Huang ^1^, Zonghua Liu ^1^, Chongjie Wu ^2^, Jiansheng Lin ^3,^ *, Ning Liu ^2,^ *

^1^ Department of Biomedical Engineering, Jinan University, Guangzhou, China, 510632

^2^ Department of Bone and Joint Surgery, The First Affiliated Hospital of Jinan University, Jinan University, Guangzhou, China, 5106323

^3^ Department of Anatomy, Hunan University of Chinese Medicine, Changsha, China, 410208

Corresponding authors:

Jiansheng Lin ((linjiansheng1020@163.com)

Ning Liu (liuning@163.com)

**Results**


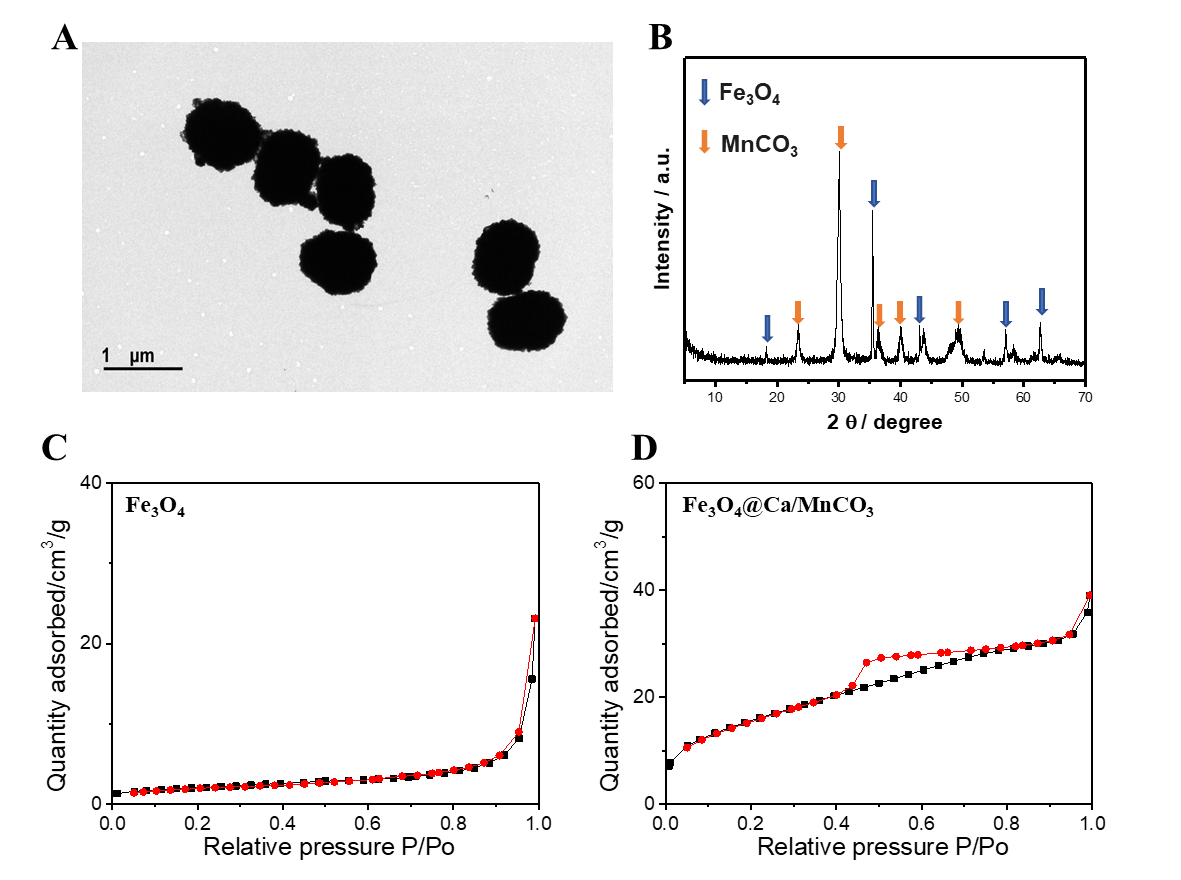


Figure S1. (A) TEM image of Fe_3_O_4_@Ca/MnCO_3_ nanoparticles. (B) The XRD spectrum of Fe_3_O_4_@Ca/MnCO_3_ nanoparticles. The nitrogen adsorption-desorption isothermal curves of Fe_3_O_4_ (C) and Fe_3_O_4_@Ca/MnCO_3_ (D) nanoparticles.


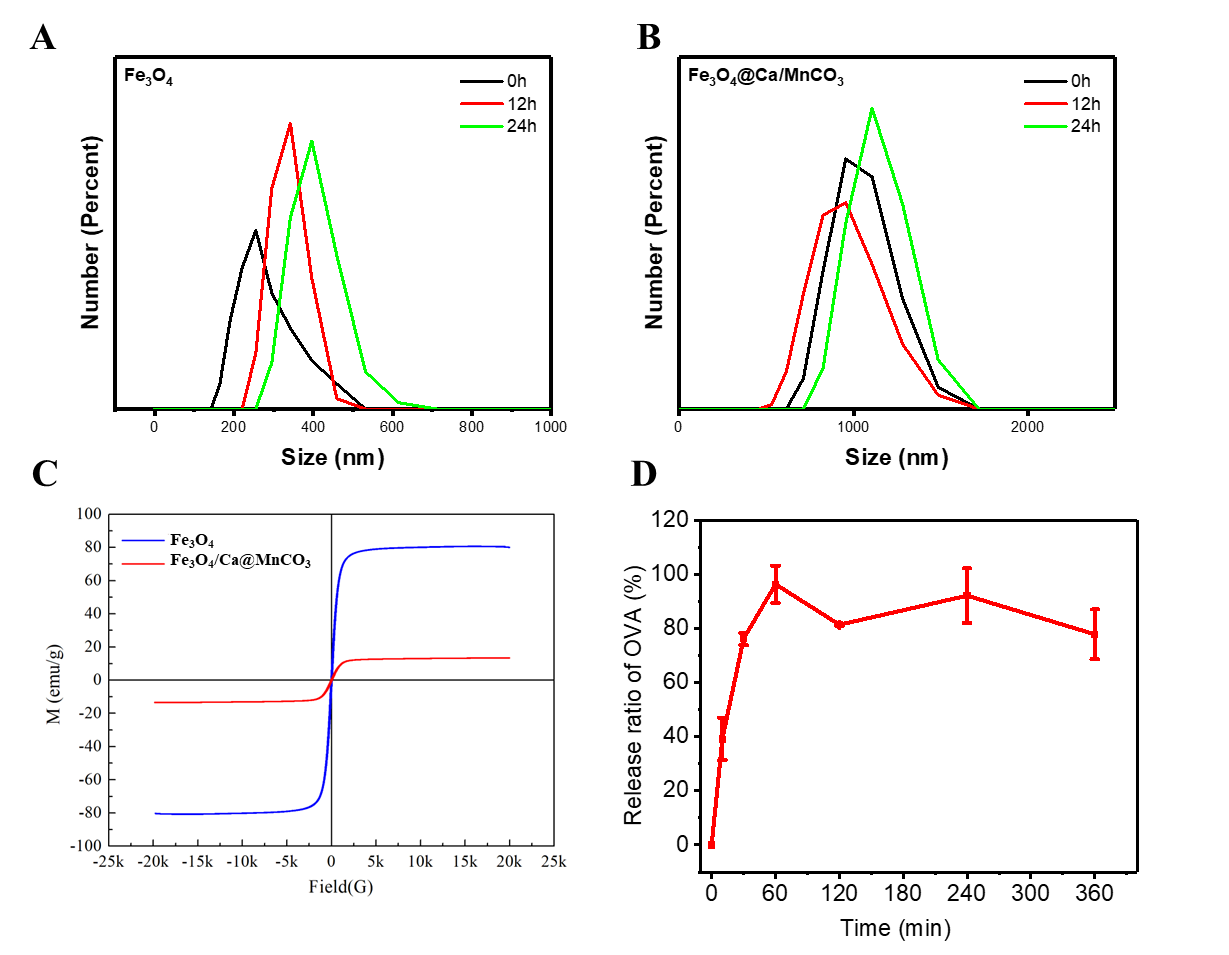


Figure S2. The sizes of Fe_3_O_4_ (A) and Fe_3_O_4_@Ca/MnCO_3_ (B) nanoparticles at different times. (C) Magnetization curves of Fe_3_O_4_ and Fe_3_O_4_@Ca/MnCO_3_ nanoparticles. (D) Release ratio of OVA in acid buffer solution (pH5.6).


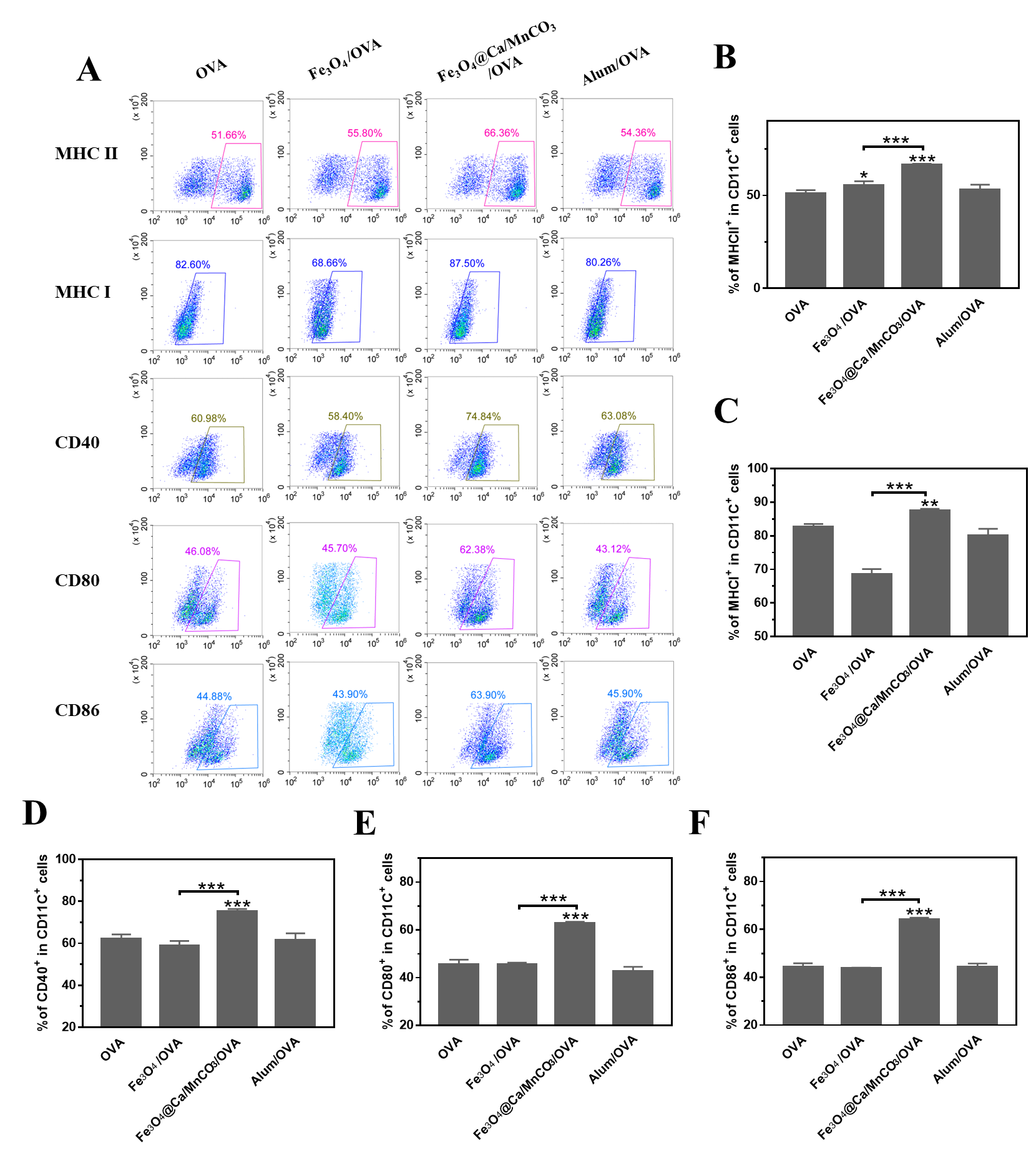


Figure S3. Maturation and antigen presentation of BMDCs induced by the nanoparticle formulations. (A) Representative scatter plots of histocompatibility complexe and costimulatory molecule expressions, and the corresponding percentages of MHC II (B), MHC I (C), CD40 (D), CD80 (E) and CD86 (F) molecules expressed on CD11c^+^ DC cells. *P<0.05, **P<0.01, ***P<0.001, compared with the OVA group.


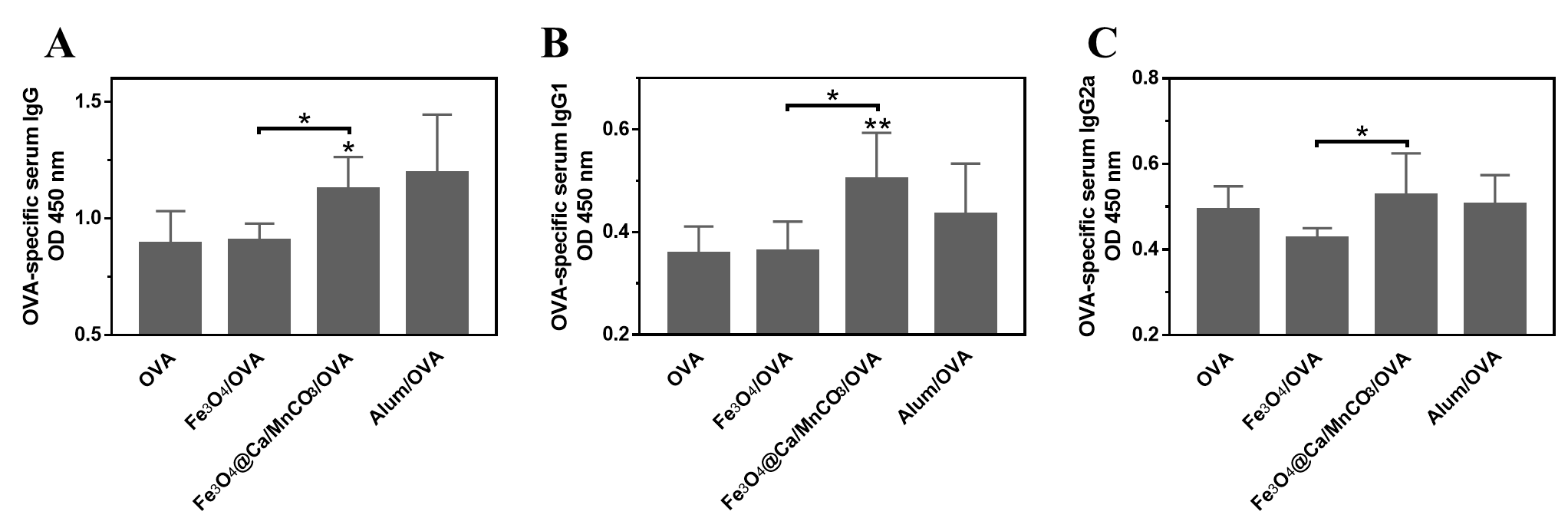


Figure S4. The mice immunized with each DC vaccine. Serum OVA specific IgG (A), IgG1 (B) and IgG2a (C) titer detected by ELISA. *P<0.05, **P<0.01, compared with the OVA group.


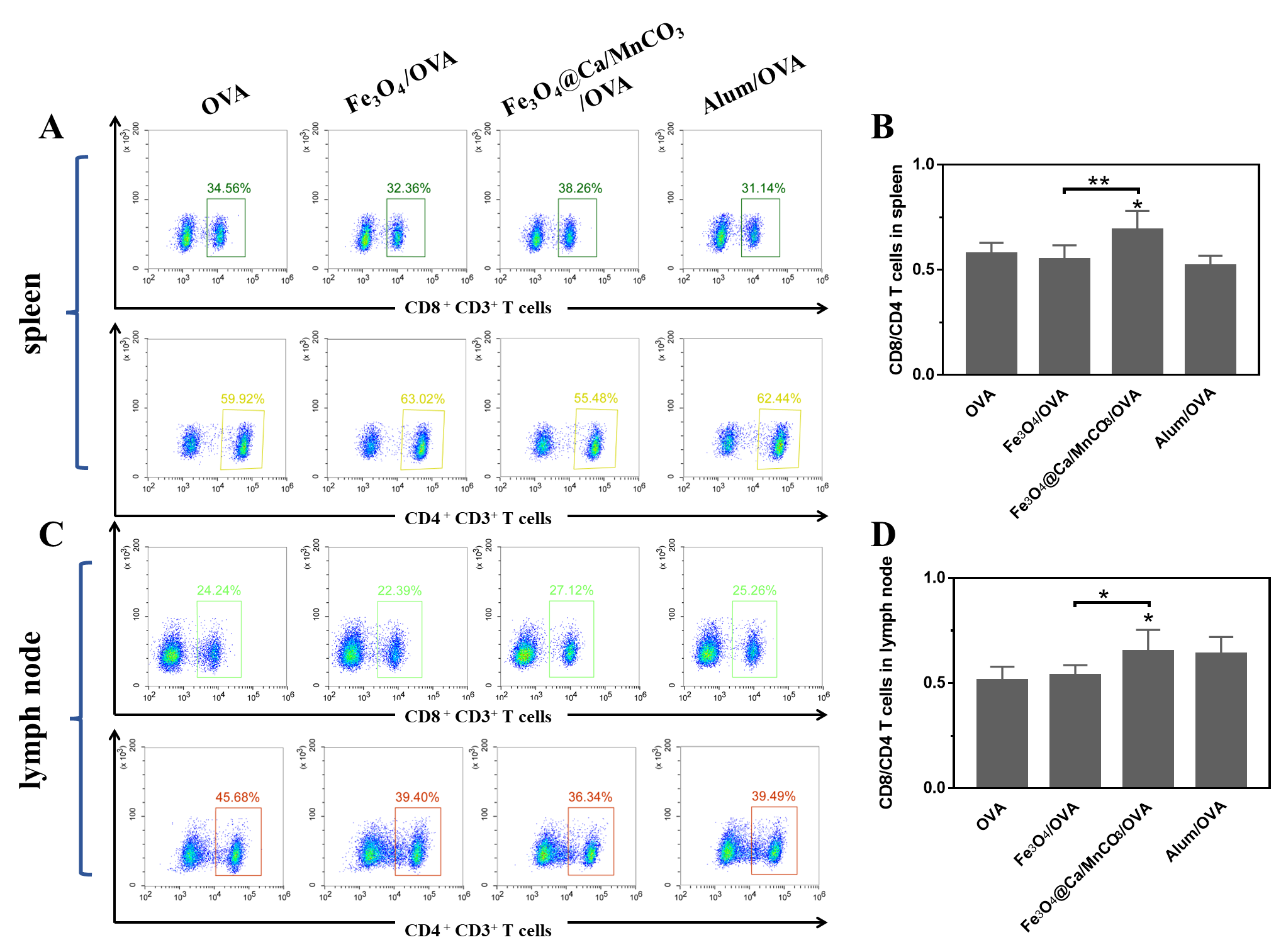


Figure S5. The mice immunized with each DC vaccine. Proportion of CD8^+^ CD3^+^ and CD4^+^ CD3^+^ T cells in splenocytes (A) and lymphocytes (C), and the ratio of CD8^+^ CD3^+^/CD4^+^ CD3^+^ T cells in splenocytes (B) and lymphocytes (D). *P<0.05, **P<0.01, compared with the OVA group.
